# Computational Model of Antidepressant Response Heterogeneity as Multi-pathway Neuroadaptation

**DOI:** 10.3389/fphar.2017.00925

**Published:** 2017-12-20

**Authors:** Mariam B. Camacho, Thomas J. Anastasio

**Affiliations:** ^1^Computational Neurobiology Laboratory, Beckman Institute for Advanced Science and Technology, Neuroscience Program, Medical Scholars Program, University of Illinois College of Medicine at Urbana-Champaign, Urbana, IL, United States; ^2^Computational Neurobiology Laboratory, Department of Molecular and Integrative Physiology, Beckman Institute for Advanced Science and Technology, University of Illinois at Urbana-Champaign, Urbana, IL, United States

**Keywords:** depression, monoamine, serotonin, norepinephrine, dopamine, SSRI, systems biology, neural network

## Abstract

Current hypotheses cannot fully explain the clinically observed heterogeneity in antidepressant response. The therapeutic latency of antidepressants suggests that therapeutic outcomes are achieved not by the acute effects of the drugs, but rather by the homeostatic changes that occur as the brain adapts to their chronic administration. We present a computational model that represents the known interactions between the monoaminergic neurotransmitter-producing brain regions and associated non-monoaminergic neurotransmitter systems, and use the model to explore the possible ways in which the brain can homeostatically adjust to chronic antidepressant administration. The model also represents the neuron-specific neurotransmitter receptors that are known to adjust their strengths (expressions or sensitivities) in response to chronic antidepressant administration, and neuroadaptation in the model occurs through sequential adjustments in these receptor strengths. The main result is that the model can reach similar levels of adaptation to chronic administration of the same antidepressant drug or combination along many different pathways, arriving correspondingly at many different receptor strength configurations, but not all of those adapted configurations are also associated with therapeutic elevations in monoamine levels. When expressed as the percentage of adapted configurations that are also associated with elevations in one or more of the monoamines, our modeling results largely agree with the percentage efficacy rates of antidepressants and antidepressant combinations observed in clinical trials. Our neuroadaptation model provides an explanation for the clinical reports of heterogeneous outcomes among patients chronically administered the same antidepressant drug regimen.

## Introduction

Depression is a serious neuropsychiatric disorder lacking effective pharmacological interventions. The first-line antidepressant medications in use today are selective serotonin reuptake inhibitors (SSRIs) (Celada et al., 2004; Koenig and Thase, 2009). Second-line treatments include augmentation of an SSRI with an atypical antipsychotic or another drug that targets the monoaminergic neurotransmitter systems [serotonin (5HT), norepinephrine (NE), or dopamine (DA)] (Boulton et al., 2010; Han et al., 2013). Unfortunately, only two thirds of depressed patients improve with initial SSRI treatment and only one third experience complete remission of symptoms (Stewart et al., 2014). These unideal efficacy rates, which indicate antidepressant response heterogeneity, are persistent problems in the treatment of depression. Better understanding of antidepressant mechanisms of action is needed.

The majority of antidepressants in use today were developed on the basis of the monoamine hypothesis, which is consistent with findings that effective antidepressant drugs enhance monoamine neurotransmission, and posits that elevated monoamines alleviate depression (Schildkraut, 1965). The current understanding of SSRI mechanism is derived primarily from rodent studies, as similar acute and chronic pharmacological effects of antidepressants have been observed in rodents and humans [(Berney et al., 2008; Gray et al., 2013) reviewed in (Dulawa and Hen, 2005)]. Acutely, SSRIs inhibit the 5HT transporter protein and increase 5HT in the extracellular space (Romero et al., 2003). This activates the somatodendritic 5HT1A autoreceptor on dorsal raphe nucleus (DR) 5HT-producing neurons (Artigas et al., 1996). A decreased firing rate of 5HT neurons results, causing a subsequent decrease in 5HT release (de Montigny et al., 1990). The net effect of decreased reuptake due to SSRI and decreased DR firing rate due to inhibition via 5HT1A autoreceptors is an increase in extracellular 5HT of 200% or more, depending on the SSRI (Bymaster et al., 2002). However, the acute rise in 5HT is not enough to cause relief from depressive symptomology (Artigas et al., 1994). Four to 6 weeks of maintained SSRI is required to achieve an antidepressant effect. Under chronic SSRI in rats, 5HT neuron firing rate returns to normal following desensitization of DR 5HT1A autoreceptors (de Montigny et al., 1990). This restoration of DR 5HT neuron firing activity coupled with 5HT reuptake inhibition (due to maintained SSRI) leads to greatly increased 5HT release. With chronic SSRI treatment, extracellular 5HT rises above 400% of baseline in rats and is associated with depression relief (de Montigny et al., 1990; Ceglia et al., 2004). These findings led to the view that chronic SSRIs work by blocking 5HT reuptake and by causing desensitization of DR 5HT1A autoreceptors, which greatly elevates 5HT levels, and in turn results in an antidepressant effect.

A number of questions remain unanswered in the current view. The acute rise in extracellular 5HT should expose all 5HT receptor types to elevated 5HT. Findings in rats indicate that most 5HT1A receptors located on non-DR neurons (5HT heteroreceptors) do not desensitize with SSRI treatment (reviewed in Blier and de Montigny, 1994). Why don’t all 5HT1A receptors desensitize equally? Why is it mainly the DR 5HT1A autoreceptors that desensitize? And why is the SSRI efficacy rate so low?

To try to answer these questions, we considered the whole monoaminergic neurotransmitter system and took into account the following findings: Antidepressants that have been studied neurophysiologically cause changes in the firing rates of the neurons in at least one of the monoamine-producing nuclei [DR produces 5HT, locus coeruleus (LC) produces NE, and ventral tegmental area (VTA) produces DA], and many cause firing-rate changes in two or even all three nuclei [(Katz et al., 2010; Ghanbari et al., 2012; El Mansari et al., 2015; Oosterhof et al., 2015), reviewed in (Blier and El Mansari, 2013)]. The same neurons that secrete the monoaminergic neurotransmitters co-secrete other neurotransmitters and neuropeptides (Studler et al., 1985; Melander et al., 1986). Drug-induced changes in DR, LC, and VTA firing rate would alter secretions of the monoaminergic and some non-monoaminergic transmitters, and this would alter the activities of neurons in the monoaminergic nuclei and also in the other brain regions with which they interact.

Neuroadaptation experiments have shown that neurons will adjust the strength of their inputs in order to restore normative firing rates, specifically by increasing or decreasing the sensitivity or expression of receptors (Turrigiano, 2012). This normalization process can require multiple days (Turrigiano and Nelson, 2000). The concept of neuroadaptation is central to our current understanding of the mechanism of action of antidepressant drugs (de Montigny et al., 1990; Hensler, 2003). It is likely that neuroadaptive processes come into play under chronic antidepressant administration and that SSRIs become effective only after a period of neuroadaptation.

We simulated neuroadaptation in a model in which antidepressant administration could change the firing rates of neurons in the monoaminergic nuclei (represented as the activations of model units), and adjustments in receptor strength could bring those activations back toward no-drug baselines. We show that different adjustment pathways can reach different receptor strength configurations that can achieve similar levels of neuroadaptation but with different adapted levels of production of the three monoamines. Since monoaminergic neurotransmitter levels are related to therapeutic efficacy (Blier and de Montigny, 1994; Rush et al., 2006), between-individual differences in the production of the three monoamines could explain why some individuals experience a therapeutic benefit with a chronic antidepressant while others taking the same drug do not. The model answers questions left open by the current view of SSRI mechanism, provides insight into possible pharmacological treatments for different subtypes of depression, and makes an experimentally testable prediction of broad potential relevance.

## Materials and Methods

The model is based on a homeostatic hypothesis, whereby the units in a neural network representing the interactions between the monoaminergic nuclei can modulate their input levels by adjusting their neurotransmitter receptor strengths. These adjustments can be neuroadaptive under chronic antidepressant in that they can bring the activation levels of the units back toward their normative, no-drug baselines. In order to exploit the complementary capabilities of two different programming modalities, we implemented the same model in two computer programs written in two very different languages. One programming language is imperative (MATLAB^®^), while the other is declarative (Maude) (Supplementary Section S1). We used MATLAB to optimize the parameters of the model and to bring its behavior in line with observation, and we used Maude to search the space of possible combinations of model receptor strengths and to determine whether neuroadaptation to chronic antidepressant could be achieved with different receptor strength configurations.

### Model Structure and Function

The model is an abstract representation of the three main monoaminergic neurotransmitter systems, along with three related non-monoaminergic neurotransmitter systems whose interactions with the monoaminergic systems have been well described. It takes the form of a recurrent network in which the three monoaminergic brain regions (DR, LC, and VTA), as well as three non-monoaminergic neurotransmitter systems [corticotropin-releasing factor (CRF), galanin, and glutamate], are each represented as single, non-linear model units. The monoaminergic regions are represented because they have been implicated in studies of depression and mood through the monoamine hypothesis (Schildkraut, 1965; Coppen, 1967). The galanin and glutamate transmitter systems are represented because these transmitters can be co-secreted along with the monoamines (Melander et al., 1986; Gagnon and Parent, 2014; Liu et al., 2014). The CRF system is included because CRF receptors have been shown to change their expression levels in the DR in response to stress and chronic antidepressant use (Fernandez Macedo et al., 2013).

The recurrent network model is composed of six non-linear units that represent the three monoaminergic brain regions (DR, LC, and VTA) and the three non-monoaminergic neurotransmitter systems (CRF, galanin, and glutamate). Each unit projects to every other unit including itself. The strengths of the projections of the non-monoaminergic units onto themselves, between each other, and from the monoaminergic units are represented as generic weights. The non-monoaminergic units each release only one transmitter onto the monoaminergic units, which is the transmitter they represent in the model. While VTA releases only DA onto the other monoaminergic units, DR and LC release 5HT and NE, respectively, as well as other transmitters onto the other monoaminergic units. Such release and co-release has been described in the literature (Supplementary Section S2; see also **Table 1**).

**Table 1:** Transmitters, receptors, and connection weights mediating interactions between model units.

From	DR	LC	VTA	tCRF	Tgal	Tglu
(across)	*5HT*	*NE*	*DA*	*CRF*	*gal*	*glu*
To	*gal*	*gal*				
(down)	*glu*					
**DR**	5HT1AR(-)	AR1(+)	D2R(+)	CRF1R(-)	galR1(-)	AMPAR(+)
		galR1(-)		CRF2R(+)	galR2(+)	
		galR2(+)				
**LC**	AMPAR(+)	AR2(-)	D1R(-)	CRF1R(+)	galR1(-)	AMPAR(+)
	5HT2AR(-)		D2R(+)			
	galR1(-)					
**VTA**	AMPAR(+)	AR1(+)	D2R(-)	CRF1R(+)	galR1(-)	AMPAR(+)
	5HT2AR(+)	AR2(-)				
	5HT2CR(-)	galR1(-)				
**tCRF**	wDR(+/-)	wLC(+/-)	wVTA(+/-)	wCRF(+)	wGal(+/-)	wGlu(+/-)
**Tgal**	wDR(+/-)	wLC(+/-)	wVTA(+/-)	wCRF(+/-)	wGal(+)	wGlu(+/-)
**Tglu**	wDR(+/-)	wLC(+/-)	wVTA(+/-)	wCRF(+/-)	wGal(+/-)	wGlu(+)


*Interactions between the monoaminergic units (DR, LC, and VTA) are mediated by both excitatory (+) and inhibitory (-) receptors. A given monoaminergic unit is assigned one or at most two receptors for each transmitter it receives, which are the predominant receptors for the corresponding transmitter as determined through a comprehensive literature search (see text for references). Each monoaminergic unit may release more than one neurotransmitter onto another unit (listed in column headers), again as described in the literature. The non-monoaminergic units each release only one transmitter. They do not have receptors. Instead, the connections onto non-monoaminergic units from themselves, from each other, and from the monoaminergic units have generic connection weights. These weights are denoted by the letter “w” followed by a name associated with the sending unit (e.g., wDR is a connection weight from the DR unit while wGal is a connection weight from the Tgal unit). Note that all receptors and weights are unit specific (e.g., wDR takes different values onto tCRF, Tgal, and Tglu). The sign in parenthesis (+ or -) denotes the fixed polarity of a receptor or of a generic self-connection weight, while +/- means that the polarity of the corresponding connection weight is not fixed but can be set positive or negative during the parameter optimization process.*

**FIGURE 1:**
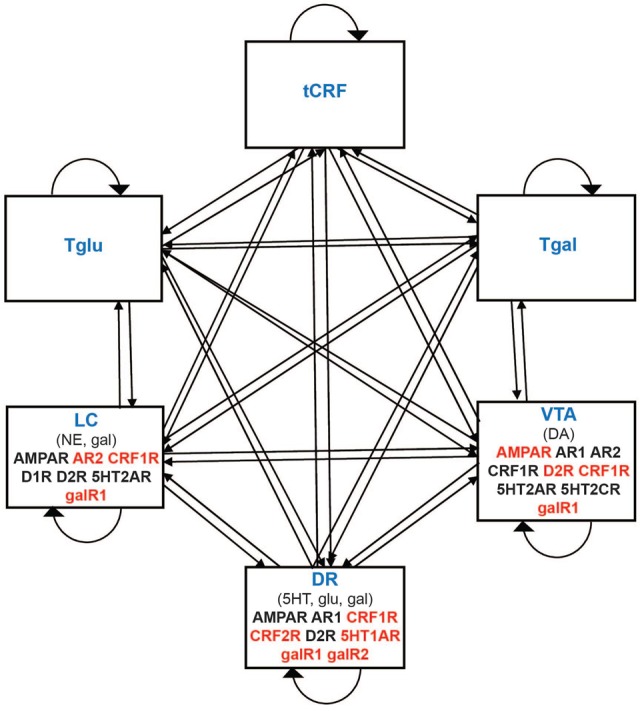
Schematic representation of the monoaminergic neurotransmitter system model. Each rectangle denotes a unit in the model that represents either a monoaminergic brain region or a set of regions that secrete a non-monoaminergic neurotransmitter. DR, LC, and VTA (monoaminergic) refer to the dorsal raphe nucleus, locus coeruleus, and ventral tegmental area, respectively. tCRF, Tgal, and Tglu (non-monoaminergic) refer to corticotrophin releasing factor (CRF), galanin, and glutamate neurotransmitter systems, respectively. Connections between model elements can be excitatory or inhibitory and take the form either of the strengths of neurotransmitter-specific receptors (onto monoaminergic units) or of generic connection weights (connections onto non-monoaminergic units). Receptors mediating the predominant effect of each transmitter on the monoaminergic brain regions are denoted in their respective rectangles. Adjustable receptors are represented in red.

We define the predominant receptor type as that type which mediates the main effect of a specific transmitter on a specific neural type (e.g., the predominant receptor type for NE on DR neurons is the α1-adrenergic receptor). The predominant receptor types on DR, LC, and VTA neurons for the six transmitters represented in the model have been well described (Supplementary Section S2), and the connections onto the monoaminergic units in the model are implemented using the predominant receptors specific for each of the different transmitters released onto them (**Figure 1** and **Table 1**). Some of these receptors are known to be adaptable with antidepressant drugs (see below). The connections onto the non-monoaminergic units in the model are implemented using generic connection weights, because each non-monoaminergic unit represents a heterogeneous set of neural types that have in common only that they all release the corresponding non-monoaminergic transmitter, and so the idea of a predominant receptor type on a specific neural type does not apply. Findings on the interactions between monoaminergic regions were compiled via comprehensive literature search (Supplementary Section S2). **Figure 1** summarizes these interactions graphically. **Table 1** shows the transmitter(s) released by each unit and the receptors or generic connection weights each bears.

Each unit releases its transmitter(s) in an amount proportional to its activation level. For 5HT, NE, and DA, this amount is reduced by the transporter for that transmitter. In addition to its receptor or generic weight strengths, each unit also has a bias parameter. Each unit computes the sum of its inputs, whether due to receptors (monoaminergic units) or generic weights (non-monoaminergic units), adds its bias, and passes the result through the sigmoidal squashing function. The squashing function bounds the activations of the units non-linearly but smoothly between 0 and 1 (Supplementary Section S2). To evaluate unit activity with or without drugs, the activity of all units is set to 0, and the units are allowed to influence each other’s activity for 150 time steps. Due to the inhibitory self-connections of the monoaminergic units (self-inhibition via autoreceptors) and asymmetries in the connections between units (Supplementary Section S2), the activities of the units in the model are prone to alternate and oscillate (Luenberger, 1979). Following an initial transient, the running average of the oscillations of all units settles down to a constant value within 50 time steps (see Results). The activation of any unit is taken as the running average of its activity over the second half of the time step range (between time steps 75 and 150 inclusive).

### Setting Model Parameters

The parameters of the model are the receptor strengths, generic weights, and the bias levels already mentioned, as well as the efficacies of the monoaminergic transmitter transporters and the strengths of various drugs on their targets. These parameters were optimized using the genetic algorithm (GA) as implemented in MATLAB (Supplementary Section S3). Rather than maximize a fitness function, we used the GA to minimize an error function. The error function provided a measure of the difference between the behavior of the monoaminergic units in the model and that of real monoaminergic neurons in their responses to acute administration of various drugs. Data used as the targets of optimizations were derived from the work of Pierre Blier. The Blier lab studied acute (2-day) and chronic (14-day) antidepressant drug effects in male Sprague-Dawley rats using subcutaneous osmotic mini-pumps. Single-unit recordings from presumed 5HT, NE, and DA neurons in the DR, LC, and VTA, respectively, and others such as hippocampal CA3 neurons were made after 2 and 14 days. The error function includes data on the eight drugs and two drug combinations as studied by the Blier lab using this protocol. The eight drugs and drug pairs are: Escitalopram, Nomifensine, Reboxetine, Trazodone, Asenapine, Aripiprazole, Bupropion, Quetiapine, Escitalopram/Aripiprazole, and Escitalopram/Quetiapine. For consistency in the initial model, only the drugs studied by the Blier lab under this specific protocol were included. The acute effects of these drugs were used for model parameter optimization. Descriptions of these drugs and their targets, along with references to the primary literature, are provided in Supplementary Table S1.

Through reduction of error, the GA optimized several criteria in addition to the agreement in the percentage changes in monoaminergic neuron activation levels due to acute drugs. These other criteria include the activation levels of the units in the absence of drugs, and the levels of the monoaminergic neurotransmitters in the absence of drugs or in the presence of the transporter blocking drugs Escitalopram and Reboxetine (Supplementary Section S3). Of the 200 GA searches we ran, we selected the 10 lowest-error parameter sets for further consideration.

### Selection of One of the Ten Best Parameter Vectors

Models parameterized with the 10 best parameter vectors were preliminarily evaluated to determine their adaptive capabilities using MATLAB. All 10 showed the expected rises in 5HT with acute Escitalopram (a 5HT transporter blocker) and in NE with acute Reboxetine (an NE transporter blocker), but this was already an error function criterion and does not involve receptor adaptation. Additionally, in all 10, the 5HT level, which rose with acute Escitalopram, could rise even further after adaptation to Escitalopram following receptor strength adjustments. All units in all of the 10 best parameterizations showed some oscillation.

In nine of the 10 cases, changes in adjustable receptors shifted the oscillation offset (the constant value about which the oscillation occurs). In these cases, the change in the offset was responsible for the change in the running average of the oscillation. In one case, receptor adjustments mainly changed the amplitude of the oscillation and had little effect on oscillation offset. Due to the compressive nature of the squashing function, this amplitude change produced an apparent offset shift that disrupted the more typical relationship between receptor strength and actual oscillation offset (Supplementary Section S3). Because of the complex dynamics associated with this parameterization, it was eliminated.

Of the remaining nine cases, the fit with error 9.93 was selected for further analysis. This fit was considered representative because its error fell at the high end of the 10-best root mean square (RMS) error range (from 5.66 to 9.94, see Results) but it could also produce a robust increase in 5HT due to desensitization (reduction in strength) of the 5HT1A autoreceptor on the DR unit under chronic Escitalopram, which returned DR activation back toward baseline, no-drug levels.

### Receptor Adjustment Approach

Simulated administration of a drug alters the activation levels of the units in the model. Neuroadaptation was simulated by allowing the model to adjust the strengths of a subset of its receptors, specifically those that are known to adjust to chronic antidepressant administration, in order to bring the activation levels of the monoaminergic units back toward their baseline values. The receptors that adapt in the model are both type and neuron specific. The 11 adjustable receptors are the three monoaminergic autoreceptors: 5HT1A on DR neurons, α2-adrenergic receptor (AR2) on LC neurons, and dopamine receptor D2 (D2R) on VTA neurons; and specific receptors for CRF, galanin, and glutamate: galanin 1 receptors (galR1) on DR and LC neurons, galanin 2 receptors (galR2) on DR neurons, CRF1R on DR, LC, and VTA neurons, CRF2R on DR neurons, and α-amino-3hydroxy-5-methyl-4-isoxazolepropionic acid receptors (AMPAR) on VTA neurons. The Blier group (and others) found that these receptors adjust under chronic antidepressant treatment (Blier and de Montigny, 1987; Szabo and Blier, 2001, 2002; El Mansari et al., 2008; Chernoloz et al., 2009; Ghanbari et al., 2010; Katz et al., 2010; Madhavan et al., 2013; El Iskandrani et al., 2015). The model is agnostic as to whether adaptive changes in receptor strength are due to changes in expression, sensitivity, or both.

In the model, any 1 of the 11 adjustable receptors could be adjusted up or down by 1 on each adjustment step. The MATLAB version was used for making receptor strength adjustments along single sequences (i.e., pathways) in which the receptor to be adjusted at any step was chosen at random. The adjustment was retained only if it resulted in a homeostatic reduction in activation error. The results of runs of 100 random adjustments are reported in section “Results.”

In separate computer experiments, 1,000,000 randomly ordered strictly error-reducing sequences of adjustments (any 1 of 11 receptors, adjusted either up or down by 1) were allowed to continue until further receptor strength adjustment produced no further reduction in activation error with chronic Escitalopram. We found that the majority of sequences (mode of the dataset) took 7 adjustment steps to reach complete adaptation. The number of adjustments ranged between 2 and 26 with an average of 7.9 (see Supplementary Figure S2). Because these experiments continued until error could not be further reduced, they produced terminally adapted receptor strength configurations. We plotted 1000 configurations terminally adapted to chronic Escitalopram in Supplementary Figure S3. This figure shows that there is not a single, unique, terminally adapted receptor strength configuration; in fact, there appear to be a multitude of heterogeneous configurations.

The Maude version was used for exhaustive searches of the entire space of receptor strength configurations reachable in all sequences of length 3. Maude elaborated the tree of every possible sequence of receptor adjustments where adjustments were limited only by the lower and upper receptor-strength bounds (0–10 in absolute value). For Maude searches, receptor adjustments were allowed whether or not they reduced activation error. This allowed for the possibility that certain low error configurations could be reached only after passing through higher error configurations. Exhaustive search in Maude was limited to sequences of three adjustment steps (see below).

### Details on Maude Searches

Maude generates the full set of possible receptor strength configurations by making every possible sequence of receptor strength adjustments (i.e., Maude elaborates the entire tree of possible receptor strength adjustment sequences). Maude then searches the space of all possible receptor strength configurations by searching over the tree of all receptor adjustment sequences (i.e., pathways).

Each of the 11 adjustable receptors can either increase its absolute strength by 1, or by a fraction if its strength is greater than 9 but less than 10 (the predetermined maximum receptor strength). Each receptor can either decrease its absolute strength by 1, or by a fraction if its strength is less than 1 but greater than 0. Because each of the 11 adjustable receptors can potentially increase or decrease its strength at any adjustment step (increase or decrease is not allowed for receptors already at strength 10 or 0, respectively), there are potentially 22 different adjustments that can be made at any point along any sequence (i.e., at any level in the sequence tree). The number of possible receptor strength configurations at any level can then be computed as *n^v^*, where *n* is the number of possible adjustments and *v* is the level of the tree (or position in the sequence). Specifically, level 0 has 1 receptor strength configuration, which is the initial configuration, level 1 has 22 configurations at most, level 2 has 22^2^ or 484 configurations at most, and level 3 has 22^3^ or 10,648 configurations at most, and so on. The total upper bound of possible receptor strength configurations in any sequence tree is then the sum of the possible configurations at every level. For a tree composed of sequences of length 3, for example, the total upper bound of possible receptor strength configurations is 11,155. Due to the computational overhead of determining the consequences of any change in receptor strength configuration (i.e., a change in any 1 receptor) for a network of 6 units over 150 time steps, our searches of receptor strength configuration space were limited to trees of sequence length 3 (see section “Hardware Considerations”).

We searched the sequence tree for receptor strength configurations that were adapted, in the sense that monoaminergic unit activation returned close enough to baseline to bring activation error within a specified criterion. The amount of network activity deviation from the baseline (no-drug) value due to acute drug administration in the model depended on the drug or combination in question, so the amount of reduction in activation error that qualified as “adapted” had to be set specifically for each drug or drug combination. All error values lower than the lowest error at level 1 were considered to be “adapted” at levels 2 or 3.

Among the adapted receptor strength configurations, we also searched for configurations that achieved certain levels of the monoaminergic transmitters. “Therapeutic” monoamine levels have not yet been determined unequivocally. We set our search criteria conservatively on the basis of the percentage changes in monoaminergic transmitter levels associated with reduction in depression symptomology that we were able to find in the literature. We were unable to find reports of studies directly showing efficacy of SSRIs that also measure 5HT levels in the brain. However, both preclinical and clinical evidence has accumulated to support the hypothesis that the 5HT system is involved in the therapeutic action of SSRIs and several other antidepressant drugs by elevating 5HT (reviewed in Blier and de Montigny, 1994). Specifically, one study found that chronic Escitalopram increases 5HT levels in the prefrontal cortex to 422% of baseline (Ceglia et al., 2004). Also, it has repeatedly been found that chronic SSRI use elevates 5HT levels to around 400% (Romero et al., 2003; Beyer and Cremers, 2008). We considered adapted receptor strength configurations for which 5HT was elevated by more than 400% of normal baseline to be therapeutic.

Rodents undergoing the forced swim test (which produces “behavioral despair”) that were given Reboxetine [a norepinephrine reuptake inhibitor, (NERI)] and demonstrated decreased immobility (i.e., antidepressant effect) were found using microdialysis to increase NE levels to 212% of baseline (Page et al., 2003; Can et al., 2012). Rats that demonstrated alleviation of depressive symptomatology (measured through increased locomotor activity) due to administration of St. John’s Wort were found to increase DA levels to 140% of baseline in the prefrontal cortex (Yoshitake et al., 2004). We considered adapted receptor configurations for which NE or DA were elevated by more than 200% of normal baseline to be therapeutic.

### Hardware Considerations

The most computationally intensive procedures undertaken for this analysis were GA optimizations (in MATLAB) and state-space searches (in Maude). Both of these procedures are immanently parallelizable. MATLAB has built-in options for parallelizing GA optimizations. Unfortunately, options for parallelizing Maude searches were not available. Multiple computers were used for computational analysis. All computers had dual-core, Intel-based processors but varied in number of processors, memory capacity, and operating system.

All MATLAB GA optimizations were carried out on 16-processor Intel Zeon machines with 2, 2.60 GHz cores per processor (32 cores total) and 128GB of RAM under the Windows 7 operating system. The machines were made available by the Beckman Institute Visualization Lab. A single GA optimization that evolved a population of 100, 76-element parameter sets until the change in the fitness between generations was less than a tolerance of 10^-12^ took about 3 h on those 32-core VizLab machines.

Although parallelized Maude search was not an option, we did run multiple, serial Maude searches simultaneously on cores distributed over several machines. Maude searches were run on an Intel-inside CORE i7 processor with 2, 2.69 GHz cores and 8.00 GB of RAM under the Windows 8 operating system, and on an Intel Core 2 Duo CPU processor with 2, 2.33 GHz cores and 4.00 GB of RAM under the Windows 7 operating system. Maude elaboration and search of a state-transition tree having up to 11,155 states (i.e., receptor strength configurations) took between 4 and 22 h on each core of those dual-core machines. Given the large number of optimizations and searches necessary both to establish the paradigm and then generate the actual results, these hardware constraints largely determined the complexity of the model that we could optimize (76 parameters per optimization) and the depth of the state-transition tree we could exhaustively search (to level 3 or up to 11,155 states per search).

## Results

**FIGURE 2:**
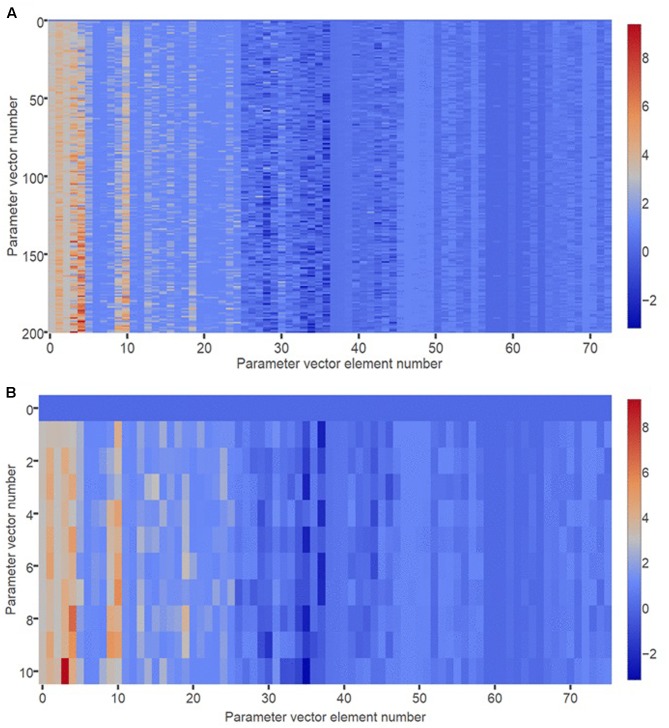
Heat map of optimized parameter vectors. **(A)** Each of 200 rows is a vector of model parameter values, optimized using the GA to produce correspondence between model behavior and observations on actual monoaminergic neurons and neurotransmitters. Each parameter vector has 76 elements corresponding to the 76 parameters of the model. All parameters take real number values. The 200 parameter vectors are ordered by Euclidian distance from a vector of all zeros, which is shown in row 0. **(B)** The “10 best” (i.e., lowest RMS error) optimized parameter vectors are plotted separately, and ordered by Euclidian distance from a vector of all zeros, which is shown in row 0.

Before the model can be used for analysis of adapted receptor strength configurations, its parameters must be set so that model behavior agrees with experimental observations on the acute effects of antidepressants that occur before receptors have adapted. The GA was used to obtain 200 sets of parameters that achieve this agreement with acute data by minimizing the RMS value of an error function (see section “Materials and Methods”). **Figure 2A** is a heat map showing the parameter sets as parameter vectors, arranged with increasing Euclidian distance from a reference vector of all zeros. The RMS errors ranged between 5.66 and 18.45 and did not change systematically with distance from the reference vector. The heat map reveals no obvious sign of clustering, suggesting that the error function does not have multiple, widely separated minima.

**Figure 2B** is a heat map showing the 10 best (lowest error) parameter vectors arranged with increasing Euclidian distance from the reference vector. The RMS errors ranged between 5.66 and 9.94. Again there is no obvious clustering. Of the 10 best fits, one was eliminated due to its aberrant adaptive behavior (see section “Materials and Methods”). The remaining nine parameterizations produced model instances that all had similar adaptive behavior that was also consistent with experimental observations. All elevated 5HT after acute Escitalopram administration (de Montigny et al., 1990; El Mansari et al., 2005), all responded to acute Escitalopram with decreased DR unit activity (de Montigny et al., 1990), and all elevated 5HT to an even higher level after adaptation to Escitalopram (de Montigny et al., 1990; Invernizzi et al., 1992; El Mansari et al., 2005). When only the DR 5HT1A autoreceptor was allowed to adapt, all desensitized this receptor to reduce activation error (de Montigny et al., 1990; Naudon et al., 2002). The fit with the RMS error of 9.93 was selected for further analysis. It was considered representative because it had an error near the top of the error range but still displayed the required adaptive capability. The model with the representative parameter set was used in the generation of all subsequent results.

### Characterizing Baseline (No-drug) Behavior

**FIGURE 3:**
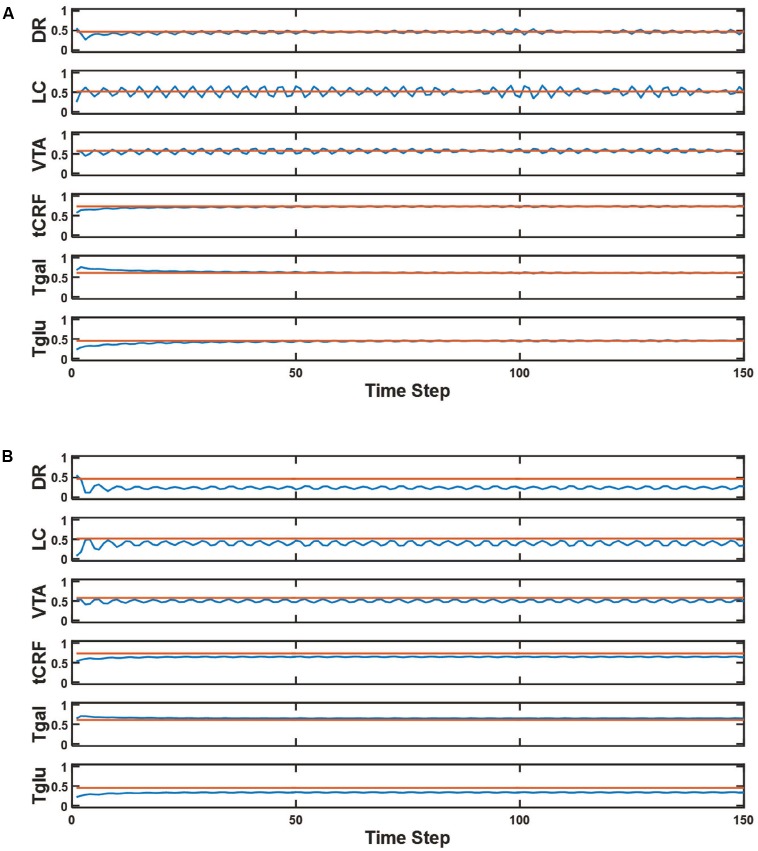
Model element activities in the baseline (no-drug) condition and acute (no adaptation) Escitalopram condition. **(A)** The blue line in each plot shows the activity of a different model unit across 150 time steps in the normal (no-drug) baseline condition. The superimposed red line is the average activity computed over the second half of the time series for the corresponding unit. These constant values for the normal condition are the baseline activations that define the normative activation of the network. **(B)** The blue line in each plot shows the activity of a different model unit across 150 time steps in the acute (no adaptation) Escitalopram condition. The red line represents the average activity of each unit at baseline, plotting the same constant value as in **(A)**. Note that Escitalopram changes the average activity level of all of the units, and especially of the DR unit.

The baseline activity of the units in the model instantiated with the representative parameter set is shown in **Figure 3A**. Following an initial transient, all units settle into a stable oscillation about a constant offset (see section “Materials and Methods”). Oscillation of unit activation was expected based on asymmetries due to known interconnections and predominant receptor-type polarities (e.g., DR inhibits LC but LC excites DR). Such complex oscillation is in qualitative agreement with observation (Gao et al., 2007; Fujisawa and Buzsaki, 2011). Neurons in the monoaminergic nuclei, and in the non-monoaminergic regions with which they interact, oscillate at multiple frequencies (ibid). We made no attempt to match model and real oscillation frequencies quantitatively; the model time-step length is arbitrary because we were interested only in average unit activations. The value of the activation of any unit under any condition (drug or no-drug) was taken as the average activity of the unit over the second half of the time series. In the no-drug case, the averaged value for each unit is the normative, baseline activation for that unit. The average activation of the DR, LC, and VTA units along with the efficacies of the 5HT, NE, and DA transporters and drugs (if present) determine, respectively, the average amount of release of 5HT, NE, and DA in the model.

The responses of this network to acute Escitalopram administration are shown in **Figure 3B**. The drug administration is acute in the sense that neuroadaptation due to receptor strength adjustment has not yet occurred. As in all drug conditions, the Escitalopram level is maintained over the course of the evaluation. Administration of Escitalopram changes the activation levels of the units including DR, LC, and VTA. This is consistent with experimental findings showing that acute administration of antidepressants can change the firing rates of neurons in those nuclei (reviewed in Blier and El Mansari, 2013, see also Supplementary Section S3).

**FIGURE 4:**
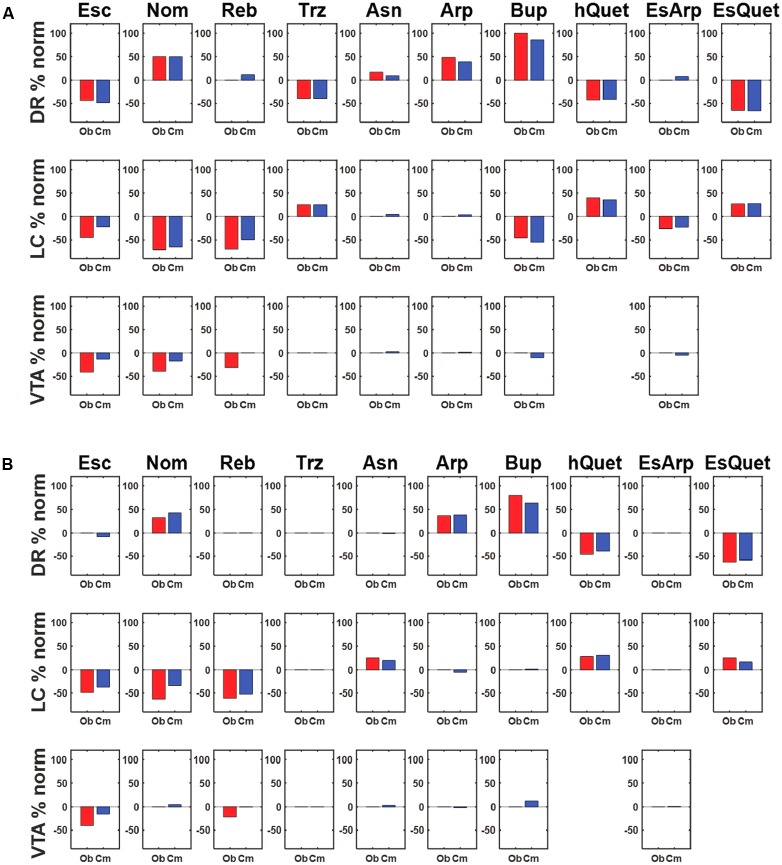
Agreement between observed and simulated percentage activation changes following acute or chronic drug administration. **(A)** Each row shows the percentage change from normal activation of the DR, LC, and VTA units, respectively, due to acute administration of the drug(s) in each column. The red bar represents the empirical value observed by the Blier lab, while the blue bar represents the computational value produced by the model. The model is parameterized using the representative parameterization (RMS error is 9.93, see text). **(B)** Each row shows the percentage change from normal activation of the DR, LC, and VTA units, respectively, in response to chronic administration of the drug(s) in each column. The red bar represents the value observed by the Blier lab. The blue bar represents the computational value produced by the model, each selected from among many adaptation runs for its agreement with the data (different runs produced different activation patterns; see text). The RMS error between the observed and computational values at the chronic stage is 9.58. Abbreviations: Escitalopram, Esc; Nomifensine, Nom; Reboxetine, Reb; Trazodone, Trz; Asenapine, Asn; Aripiprazole, Arp; Bupropion, Bup; Quetiapine, hQuet; Escitalopram + Aripiprazole, EsArp; Escitalopram + Quetiapine, EsQuet.

The activations of the monoaminergic units in the model instantiated with the representative parameter set under the acute influence of all the drugs and combinations, expressed as percentage changes from baseline, are shown in **Figure 4A** (blue bars). The percentage changes of actual DR, LC, and VTA units observed by the Blier lab under the acute influence of the same drugs and combinations (red bars) are shown for comparison. These plots demonstrate the model’s ability to reproduce the data concerning the acute effects of antidepressant drugs on neurons in the three monoaminergic nuclei. From the homeostatic viewpoint, drug-induced deviations from baseline activity can be interpreted as errors. We define the activation error as the sum of the absolute differences of the average monoaminergic unit activations from their baseline activations.

### Verifying Agreement between the MATLAB and Maude Versions of the Model

In addition to being used to optimize model parameters and for preliminary adaptation runs, the MATLAB program was used as a crosscheck for the Maude specification. Extensive crosschecking assured that the Maude and MATLAB versions of the model computed the same unit activations and transmitter levels in the no-drug and in all acute drug conditions. This validation step ensures that both versions of the model are consistent with each other and with the data on the acute effects of drugs on the activity of DR, LC, and VTA neurons. To verify agreement between the MATLAB and Maude versions, the values of multiple variables were compared after the same number of time steps (150) in both programs. We found that the activity levels of all six units (DR, LC, VTA, tCRF, Tgal, Tglu) and levels of all of the neurotransmitters were in agreement between the two programs after 150 iterations to four significant places in the no-drug condition.

We also verified that the two versions agreed on the effects of all drugs and combinations (Escitalopram, Nomifensine, Reboxetine, Trazodone, Asenapine, Aripiprazole, Bupropion, Quetiapine, Escitalopram/Aripiprazole, and Escitalopram/Quetiapine). We found agreement on the activity levels of all six units, levels of all of the neurotransmitters, and activation errors after 150 iterations to four significant places with acute administration (no-adaptation) of all of the drugs and combinations. The crosscheck strengthens confidence that the MATLAB and Maude results are uncorrupted by programming error.

### Characterizing Adaptive Behavior

To obtain a preliminary view of the adaptive capability of the model, only the DR 5HT1A autoreceptor was allowed to make adaptive adjustments. This preliminary evaluation was carried out using MATLAB, in which receptor strength adjustments are made along a single pathway (i.e., a single sequence of receptor strength adjustments). The DR 5HT1A could be adjusted up or down by 1 (or by a fraction, if its value was near the limits of 0 or 10), and the direction of adjustment on any adjustment step was chosen at random. An adjustment was retained only if it reduced activation error. These single-pathway adaptation runs show that desensitization of the DR 5HT1A autoreceptor decreases activation error and brings the monoaminergic unit activations back toward baseline. Because the GA-determined values of the autoreceptor strengths were all near 3 (3.1 for DR 5HT1A, 3.2 for LC AR2, and 3.2 for VTA D2R in the representative parameter set; Supplementary Section S3), DR 5HT1A autoreceptors could desensitize almost completely in 3 adjustment steps (3 adjustments down by 1) as part of simulated neuroadaptation. This is consistent with experimental observation on DR 5HT1AR desensitization under chronic SSRI (Blier et al., 1998; Rainer et al., 2012). An example, short MATLAB adaptation run, out to five adjustments steps, is shown in Supplementary Figure S1.

In subsequent MATLAB adaptation runs, all 11 adjustable receptors were allowed to adjust (see **Figure 4B**) but did so one at a time along a single adaptive pathway. Both the receptor and the direction of adjustment (up or down by 1, or less to reach a receptor-strength limit) were chosen at random at each adjustment step, and an adjustment was retained only if it reduced activation error. The fully adjustable model was allowed to make 100 adjustment steps (only a small number of these actually reduced activation error). Each column in **Figure 4B** shows one specific adaptation run that was chosen for its agreement with the data. These runs show that agreement between model (blue bars) and observation (red bars) on changes in DR, LC, and VTA activation due to chronic antidepressant administration can be obtained using a receptor strength adjustment scheme based on neuroadaptation.

### Exhaustive Search of Adapted States

We used exhaustive search in Maude to evaluate activation error and monoaminergic transmitter levels over all possible sequences (i.e., pathways) of 3 receptor strength adjustments, including pathways over which error could increase, producing all possible configurations of receptor strengths reachable within 3 adjustment steps. Due to limitations in computational resources, exhaustive searches in Maude were limited to sequences of 3 receptor strength adjustments (see section “Materials and Methods”). Single-sequence, random adaptation runs in MATLAB show that terminal adaptation, in which further receptor-strength adjustments cannot further reduce activation error, can occur within 2 or 3 adjustment steps but require on average about 8 adjustments of size 1 to reach terminal adaptation (Supplementary Figure S2). Importantly, using MATLAB randomly to sample the space of terminally adapted configurations shows that they vary widely rather than converge to one or a few receptor-strength configurations (Supplementary Figure S3). With 11 adjustable receptors each able to increase or decrease its strength there would be 22^8^, or over 54 billion, possible configurations reachable in 8 adjustment steps. Exhaustive search on this order in Maude was not possible but search to level 3, which was possible, is highly instructive for three reasons. First, it is unknown whether a state corresponding to terminal adaptation occurs in all mice receiving chronic antidepressant treatment for 14 days (see section “Materials and Methods”), or in all humans over the course of a clinical trial (typically 6–8 weeks). Second, the GA-determined value of the DR 5HT1A autoreceptor (as well as the other two autoreceptors) in all of the 10 best parameter vectors was just over 3, so exhaustive search to level 3 includes the 3-step, almost-complete desensitization of the DR 5HT1A autoreceptor (Supplementary Figure S1). Exhaustive search to level 3 is therefore appropriate for comparison of other adapted configurations to the configuration characterized by almost-complete DR 5HT1A autoreceptor desensitization, which is currently considered to be the main mechanism of SSRI effect (Blier et al., 1998; Gray et al., 2013). Third, exhaustive search obviates concerns over bias inherent in any kind of sampling of the configuration space, such as the sampling we did for illustrative purposes in MATLAB described above.

Because the different drugs and combinations unbalanced the network to different degrees, we considered as “adapted” any receptor strength configuration at level 2 or 3 whose activation error was lower than the lowest level 1 error. We also set “therapeutic” percentage increases in monoaminergic neurotransmitter levels according to findings from the literature. The results of the exhaustive searches in Maude are reported in **Table 2** in terms of the percentages of adapted receptor strength configurations (i.e., states) that are also associated with therapeutic elevation in one or more of the monoamines. The main finding is that there are many adapted receptor strength configurations, but not all of these configurations are also associated with elevations in the monoamines. The percentages of adapted configurations that are also associated with therapeutic elevations in one or more monoamines are taken as estimates by the model of the percentage efficacies of the corresponding drug or combination. These percentages are considerably less than 100% in most cases, translating clinically to unideal efficacies and heterogeneity among individuals in patterns of monoamine production following adaptation to the same chronic drug or drug combination.

**Table 2:** Exhaustive searches of adapted receptor strength configurations.

Row number	Drug(s)	Adapted states	Number (percentage) of adapted states with therapeutic monoamine elevation						
			
			5HT	DA	NE	5HT/DA	5HT/NE	DA/NE	5HT/DA/NE
**1**	Escitalopram	655	192 (29%)	0	0	0	0	0	0
**2**	Nomifensine	2463	2267 (92%)	2463 (100%)	1628 (66%)	2267 (92%)	1531 (62%)	1628 (66%)	1531 (62%)
**3**	Reboxetine	1185	0	0	1185 (100%)	0	0	0	0
**4**	Trazodone	296	0	30 (10%)	54 (18%)	0	0	6 (2%)	0
**5**	Asenapine	63	0	0	0	0	0	0	0
**6**	Aripiprazole	706	0	0	0	0	0	0	0
**7**	Bupropion	1972	469 (24%)	1972 (100%)	1895 (96%)	469 (24%)	469 (24%)	1895 (96%)	469 (24%)
**8**	Quetiapine	662	0	60 (9%)	662 (100%)	0	0	60 (9%)	0
**9**	Nomifensine +	2462	2265 (92%)	2462 (100%)	1628 (66%)	2265 (92%)	1531 (62%)	1628 (66%)	1531 (62%)
	Escitalopram								
**10**	Reboxetine +	1218	355 (29%)	1049 (86%)	1023 (84%)	309 (25%)	294 (24%)	889 (73%)	260 (21%)
	Escitalopram								
**11**	Trazodone +	431	0	175 (41%)	162 (38%)	0	0	73 (17%)	0
	Escitalopram								
**12**	Asenapine +	132	2 (2%)	0	74 (56%)	0	0	0	0
	Escitalopram								
**13**	Aripiprazole +	179	65 (36%)	47 (26%)	45 (25%)	31 (17%)	0	0	0
	Escitalopram								
**14**	Bupropion +	2388	2308 (97%)	2388 (100%)	1760 (74%)	2308 (97%)	1717 (72%)	1760 (74%)	1717 (72%)
	Escitalopram								
**15**	Quetiapine +	586	66 (11%)	204 (34%)	586 (100%)	0	66 (11%)	204 (34%)	0
	Escitalopram								


*The model was subjected to chronic administration of eight drugs either alone or in combination with Escitalopram, and made every possible sequence of three allowed receptor strength adjustments. Adapted states expresses the number of receptor strength configurations (i.e., states) at level 2 or 3 (i.e., after 2 or 3 receptor adjustments) that have activation error lower than the lowest level 1 error. The other columns show the number of adapted receptor strength configurations (also expressed as a percentage of the total number of adapted configurations) that are associated with therapeutic elevations in one or more of the monoaminergic neurotransmitters. The therapeutic levels are >400,>200, and >200% of baseline for 5HT, NE, and DA, respectively.*

## Discussion

Our model can reproduce experimental observations based on the hypothesis that the monoaminergic nervous system adapts to chronic drugs by adjusting specific receptor strengths so as to bring the collective activation of the three monoaminergic nuclei back toward normative levels. It can reconcile findings on the mechanism of action of SSRIs that up until now have seemed inconsistent. Specifically, chronic SSRI treatment exposes all 5HT receptors to chronically elevated levels of 5HT, leading to desensitization of DR 5HT1A autoreceptors, but not to desensitization of all other classes of 5HT receptors or even of 5HT1A heteroreceptors such as those on CA3 neurons (reviewed in Blier and El Mansari, 2013). Furthermore, DR 5HT1A autoreceptors sometimes fail to desensitize, while some non-5HT receptors have been found to change their sensitivities under chronic SSRI (Hawes et al., 2005; Kuteeva et al., 2008; Gray et al., 2013).

The model demonstrates that DR 5HT1A autoreceptor desensitization contributes effectively to neuroadaptation under chronic SSRI when this receptor alone is allowed to adjust. When all adaptable receptors are allowed to adjust, neuroadaptation in the model can occur without desensitization of other 5HT receptor types but with adjustments in non-5HT receptors, and even in some cases without DR 5HT1A autoreceptor desensitization. Maude searches reveal that only 12.5% of the receptor strength configurations adapted to chronic Escitalopram that are associated with therapeutically elevated 5HT levels also downregulate the DR 5HT1A autoreceptor. This modeling result is consistent with clinical findings that chronic SSRIs can, in some cases, produce a therapeutic elevation in 5HT without DR 5HT1A autoreceptor desensitization (Gray et al., 2013). Our model suggests that the spectrum of observed receptor strength (sensitivity or expression) changes due to chronic SSRI can be understood together as part of a larger neuroadaptive process.

### Neuroadaptation as an Explanation for Antidepressant Response Heterogeneity

A key contribution of our study is a possible explanation for the clinical finding that SSRIs are effective in less than 50% of depressed patients. Exhaustive Maude search reveals many different adapted receptor strength configurations under chronic Escitalopram (see **Table 2**). Only 29% of them are also associated with therapeutically elevated 5HT levels, and close to none of them are associated with therapeutically elevated levels of any of the other monoaminergic transmitters. This finding agrees with the low efficacy rate of 24–55% for SSRIs that has been observed clinically (Young et al., 2009; Li et al., 2014).

We take the monoamine hypothesis as a starting point due to the finding that drugs that elevate the monoamines are effective over placebo in treating depression (Turner et al., 2008). 5HT may or may not be deficient in depressed patients (O’Reardon et al., 2004). However, because relief from depressive symptomatology is associated with elevated 5HT levels (Haddjeri et al., 1998), we take that finding as a starting point for understanding the chronic effects of SSRIs. The acute data used to parameterize the model were derived from normal rats that were not subjected to stressors or other manipulations designed to evoke a depressive phenotype (Supplementary Section S3). Our approach does not assume that monoamine levels are deficient prior to antidepressant treatment, but it does assume that monoamine elevation, directly or indirectly, determines antidepressant efficacy as consistent with the monoamine hypothesis (Schildkraut, 1965; Stone et al., 2008; Andrews et al., 2015).

The leading alternative to the monoamine hypothesis posits that depression is related to a decrease in the level of hippocampal brain-derived neurotrophic factor (BDNF) leading to decreased hippocampal neurogenesis (Duman and Monteggia, 2006). However, drugs that elevate monoamine levels also elevate hippocampal BDNF (Duman et al., 1997; Coppell et al., 2003; De Foubert et al., 2004; Berger et al., 2010; Castren and Rantamaki, 2010). Other hypotheses implicate neuropeptide transmitter systems or the relative activation of interacting brain regions (Berton and Nestler, 2006). However, depression relief associated with changes in the levels of neuropeptides, or in the relative activity of brain regions, are also believed to be closely associated with elevation in monoamine levels (reviewed in Stone et al., 2008, see also Supplementary Section S3). A recent alternative hypothesis suggests that depression results from alterations in receptor activity due to formation of homoreceptor and heteroreceptor complexes involving receptor types including 5HT1A, 5HT7, galR1, and fibroblast growth factor receptor 1 (FGFR1) (Borroto-Escuela et al., 2010, 2012; Renner et al., 2012). It is also possible that adaptation to chronic antidepressant also involves changes in receptor activation due to receptor complex formation (Borroto-Escuela et al., 2017). However they occur, changes in receptor strength will most likely cause changes in transmitter levels, and we assume that therapeutically elevated monoamines alleviate depressive symptomatology through associations with mechanisms that have been suggested previously, such as activation patterns favoring cortical over limbic structures, increased hippocampal neurogenesis, and others (Mayberg et al., 2000; Surget et al., 2008).

### Potential Clinical Relevance of Modeling Results on Antidepressants and Combinations

The potential clinical relevance of the modeling results is evaluated on the basis of the idea that depression subtypes exist and can be categorized as psychotic or melancholic depression, or as heterogeneous, non-melancholic depressive disorder (Parker, 2000). Patients with psychotic depression have consistently been found to respond to antidepressant drug combinations that chronically elevate DA levels, while patients with melancholic depression have shown considerable improvement with interventions that elevate NE levels (Spiker et al., 1985; Parker et al., 1992; Guelfi et al., 1995; Kelly and Cooper, 1997). Due to the finding that enhancing 5HT neurotransmission has been found to alleviate depressive symptomatology in patients with heterogeneous pathology not classically associated with either psychotic or melancholic depression, it has been proposed that serotonergic dysfunction may explain heterogeneous, non-melancholic depression (Malhi et al., 2002, 2005).

Although we focus on SSRIs (e.g., Escitalopram), our model also provides potential insights into the clinical efficacies of some antidepressant drugs other than Escitalopram, and combinations of other drugs with Escitalopram. The selective NE and DA releaser Bupropion, for example, has been shown to be at least as effective as SSRIs in clinical studies (Thase et al., 2005; Papakostas et al., 2007). In the model, Bupropion does not therapeutically elevate 5HT in as high a percentage of adapted configurations as Escitalopram (24% for Bupropion alone, 29% for Escitalopram alone), but Bupropion therapeutically elevates both NE and DA in 96% of adapted receptor strength configurations. Thus, our model suggests that Escitalopram would be more effective in cases where 5HT alone is deficient (non-melancholic depression), but that Bupropion would be most effective in cases where NE or DA is deficient (melancholic and psychotic depressions, respectively). The model suggests that Escitalopram or Bupropion alone achieve their main therapeutic effects through elevation in 5HT, or NE and DA, respectively. This is consistent with observations that 5HT depletion, but not NE depletion, cause relapse following chronic Escitalopram and that NE depletion, but not 5HT depletion, cause relapse following chronic Bupropion (Cooper et al., 1994; Evans et al., 2002).

The therapeutic efficacy of the combination of Escitalopram and Bupropion has not been rigorously studied with placebo controls, but some depressed patients that did not respond to SSRIs showed significant improvement with this combination (Leuchter et al., 2008). In the model, almost all receptor strength configurations adapted to chronic Escitalopram/Bupropion are associated with elevated levels of one or more of the monoamines, with all three monoamines elevated in 72% of these configurations. This finding offers the prediction that the combination of Escitalopram and Bupropion would exhibit high efficacy in patient populations comprising more than one depressive subtype.

Nomifensine was found to be an effective antidepressant before its use was discontinued due to the development of hemolytic anemia in patients (Brogden et al., 1979; Mueller-Eckhardt et al., 1988). Because 62% of adapted receptor strength configurations in our model therapeutically elevate all three monoamines with chronic Nomifensine, this drug could be efficacious in depressed patients deficient in any of the three monoamines. When Nomifensine and Escitalopram are combined, the monoamine profile we obtained from the model is very similar to Nomifensine by itself. This was expected because Nomifensine also targets the 5HT transporter (Brogden et al., 1979; Tatsumi et al., 1997). The model therefore suggests that Nomifensine by itself would be an effective antidepressant, but the use of this drug clinically has been discontinued (Mueller-Eckhardt et al., 1988). Development of a drug with a mechanism of action similar to Nomifensine but with fewer side effects could be useful for a broader range of depressed patients.

Reboxetine has previously been shown to alleviate depressive symptomatology by elevating NE levels with chronic use, but these findings have been contested by a meta-analysis that includes unpublished clinical trials (Page and Lucki, 2002; Page et al., 2003; Hajos et al., 2004; Eyding et al., 2010). When the model was allowed to adapt to Reboxetine, NE was the only monoamine that was significantly elevated, and reached therapeutic levels in 100% of adaptive configurations. This is in agreement with previous findings that chronic Reboxetine is associated with elevated NE levels (Hajos et al., 2004). However, antidepressants acting selectively on one monoamine, such as Reboxetine, alleviate symptoms of depression in a limited percentage of patients (Kornstein et al., 2009; Eyding et al., 2010), suggesting that elevating NE alone may not be sufficient to alleviate depressive symptomatology in most depressed patients. The combination of Escitalopram and Reboxetine has been studied in clinical trials with limited placebo controls, and it was found that the combination reduced depressive symptomatology in some SSRI non-responders (Rubio et al., 2004). We hypothesize that this clinical outcome could result because combining Escitalopram and Reboxetine increases the number of adapted states in which NE as well as 5HT levels are therapeutically elevated, providing relief to patients whose depression involves deficient NE and/or 5HT.

When the model adapted to chronic Trazodone, 10% of adapted configurations had elevated DA and 18% had elevated NE, suggesting that this drug should ameliorate depression in some cases. Trazodone has previously been shown to significantly improve depressive symptomatology over placebo in depressed patients (Cunningham et al., 1994; Weisler et al., 1994). Some authors posit this occurs by improving sleep quality (Weisler et al., 1994), suggesting that its antidepressant effect may be secondary to its anti-insomnia effect and may not directly involve monoamine elevation. The anti-insomnia effect is thought to be mediated by the effects of Trazodone on histamine receptors (Stahl, 2009). Histamine was excluded from the model because it is not co-released by any monoaminergic neurons and because we found no evidence that histamine receptors adjust their levels with chronic antidepressant administration. Trazodone itself was included in our drug corpus because, as a multi-target drug, it affects the 5HT1A, AR1, and AR2 receptors as well as the serotonin reuptake transporter (SERT) (Krege et al., 2000; Stahl, 2009), which are represented in the initial model. Data on trazodone was included mainly to expand the dataset that was used to parameterize the model.

Asenapine and Aripiprazole are antipsychotic drugs that have not been studied by themselves in clinical studies of unipolar depression. Chronic administration of these drugs produce very few adapted configurations that are associated with therapeutically elevated levels of any of the monoamines in the model, suggesting that Asenapine or Aripiprazole, administered by themselves, would not be clinically effective for depressed patients. In the model, 56% of receptor strength configurations adapted to chronic, combined Escitalopram and Asenapine were associated with therapeutically elevated NE levels. The clinical efficacy of the combination of Escitalopram and Asenapine in depression has not yet been studied, and our model suggests that this combination may have significant therapeutic efficacy in patients whose depression involves deficiencies in NE. The combination of Escitalopram and Aripiprazole therapeutically elevated 5HT levels in 36% of adapted receptor strength configurations in the model, which is somewhat higher than the 29% found with Escitalopram by itself. The model also found that 17% of configurations adapted to the combination of Escitalopram and Aripiprazole elevate both 5HT and DA, whereas no configurations adapted to Escitalopram alone elevate DA. This modeling result suggests that the combination of Escitalopram and Aripiprazole may help alleviate depressive symptomatology in patients whose depression involves deficiencies in either 5HT or DA, and agrees with findings that this combination is effective in some depressed patients who do not respond to SSRIs (Marcus et al., 2008; Matthews et al., 2009; Boulton et al., 2010; Han et al., 2013).

Quetiapine (an antipsychotic) by itself has recently been shown to ameliorate depressive symptomatology in some patients in its extended release form (Cutler et al., 2009). In our model, Quetiapine does not therapeutically elevate 5HT in any configuration, but it does therapeutically elevate NE in all adapted configurations. Importantly, one of the observed effects of Quetiapine is to elevate DA levels (Werkman et al., 2004). Only 9% of the receptor configurations adapted to Quetiapine by itself elevated DA to therapeutic levels in the model, suggesting that Quetiapine by itself can ameliorate depressive symptomatology in cases where NE is deficient and in some cases where DA is deficient. Although studies to date have not had adequate controls, the combination of an SSRI and Quetiapine appears to afford some improvement in patients that did not respond to SSRIs (Adson et al., 2004; Baune et al., 2007). When the model adapted to chronic Escitalopram/Quetiapine, 100% of adapted configurations elevate NE to therapeutic levels, 34% elevate DA to therapeutic levels, and 11% elevate 5HT to therapeutic levels. The model found that 34% of adapted receptor strength configurations had therapeutic elevations in both DA and NE. This combination could be effective in depressive subtypes that involve a deficiency of DA and NE, and in some cases where 5HT is deficient.

### Broader Implications

In that it represents all three monoaminergic regions (and some related systems), our model offers a more complete representation than other models (Supplementary Section S4). Our model is the first to represent and integrate factors including neural activities, neurotransmitter levels, receptor activations, transporter actions, and the effects of drugs on receptors and/or transporters. It introduces declarative programming and exhaustive search methods to the neuropharmacology field, and shows how the clinical efficacy rates of chronic antidepressant drugs could be related to the percentages of adapted receptor strength configurations that are associated with high levels of production of one or more monoaminergic neurotransmitters in a computational model.

The most interesting properties of the model are that the units representing the monoaminergic nuclei have many options available for adapting to chronic antidepressants, and that adapted networks vary in their patterns of release of the three monoaminergic neurotransmitters. It is crucial to point out that the activities of real neurons in the three monoaminergic brain regions do not all return to their individual, normative baselines under chronic administration of most antidepressant drugs or drug combinations. This is apparent from the experimental data summarized in **Figure 4B**, where the red bars show the percentage change from baseline in the activities of real DR, LC, and VTA neurons due to chronic antidepressant. These findings suggest that the three monoaminergic nuclei adapt collectively, rather than individually, and reach a “compromise” among the three in terms of their activations and transmitter release levels. The model demonstrates how the monoaminergic systems of different humans could follow different neuroadaptive pathways and may not all reach the same compromise under chronic administration of the same antidepressant, especially if they differ realistically in genetic makeup, prior experience, living conditions, and in other ways, thus accounting for the clinical heterogeneity in the response to antidepressants.

The model makes the general prediction that the monoaminergic systems of individuals of the same species, especially if they differ genetically or in previous experience or in environmental conditions or in other relevant ways, should differ in the patterns of activation of the three monoaminergic nuclei (DR, LC, and VTA) and of release of the three monoaminergic neurotransmitters (5HT, NE, and DA) under chronic administration of the same antidepressant drug or drug combination. Although genetic and environmental associations between monoamine levels, depression susceptibility, and antidepressant response have been explored (Rogers et al., 2004; Walf and Frye, 2010), experiments designed directly to test this general prediction have not been undertaken but would be needed to validate our modeling approach. The null hypothesis is that the monoaminergic systems of individuals of the same species will all attain the same patterns of activation of the three monoaminergic nuclei and release of the three monoaminergic neurotransmitters under chronic administration of the same antidepressant drug or drug combination, regardless of differences in strain or prior experience or environmental conditions or in other relevant factors. Verification of this null hypothesis would be of immense value in itself.

## Author Contributions

MC performed the literature searches, wrote the computer programs, performed all computer optimizations, simulations, and analysis, collected all the data, and wrote the manuscript. TA designed the study, developed the methodology, wrote the initial computer programs, directed the research, and co-wrote the manuscript.

## Conflict of Interest Statement

The authors declare that the research was conducted in the absence of any commercial or financial relationships that could be construed as a potential conflict of interest.
