# Supplementary material for: Computational Model of Antidepressant Response Heterogeneity as Multi-pathway Neuroadaptation
Source: Front Pharmacol. 2017 Dec 20;8:925. doi: 10.3389/fphar.2017.00925 (PMC5770730; doi:10.3389/fphar.2017.00925)
Supplement: Supplementary file 1 [file Data_Sheet_1.DOCX]

Supplementary Material

Antidepressant Response Heterogeneity on Multi-pathway Neuroadaptation

Mariam Bonyadi Camacho, Thomas J Anastasio*

*** Correspondence:** Thomas J Anastasio: [tja@illinois.edu](mailto:tja@illinois.edu)

# Supplementary Data

This text supplements the text in the article entitled **Computational Model of Antidepressant Response Heterogeneity as Multi-pathway Neuroadaptation**. It is meant to provide important details that are nevertheless inessential for understanding the main points.

**S1: Difference between imperative and declarative programming**

Imperative and declarative programming languages are fundamentally different and provide complementary functionality. Specifically, imperative languages are designed for writing programs that compute efficiently along a single processing stream while declarative languages are designed for writing programs that can be used to evaluate the consequences of processing along many different streams. Imperative and declarative programs differ mainly in the nature of their statements.

A statement in an imperative language is a command (eg, add three and three to get six) that is meant to be executed at a pre-specified point relative to the execution of other commands. A statement in a declarative language is a declaration of a fact (eg, three and three can be combined to get six) that may be executed as a command at various points relative to the execution of other declarations. A declarative program can be written so that certain declarations execute in all possible orders, thereby elaborating the entire space of possible consequences of executions of those declarations. In the Maude version of the model of the monoaminergic neurotransmitter systems, the declarations that specified receptor strength adjustments were allowed to execute in all possible orders. In this way, Maude evaluated the consequences for unit activations and monoaminergic transmitter levels of every possible combination of receptor strengths (receptor strength configurations) that were reachable given the allowed number of adjustments.

**S2: Details on model structure and function**

The monoaminergic units have receptors for each transmitter type they receive from themselves and from other units. The specific receptors represented in the model are those that mediate the predominant effect of each transmitter as described in the literature. The monoaminergic regions, DR, LC, and VTA, have been found to project directly to one another ([Beckstead, Domesick et al. 1979](#_ENREF_6), [Ornstein, Milon et al. 1987](#_ENREF_59), [Kalen, Skagerberg et al. 1988](#_ENREF_40), [Guiard, El Mansari et al. 2008](#_ENREF_33)). DR inhibits LC and VTA through 5HT2A and 5HT2C receptors (5HT2AR, 5HT2CR), respectively ([Pessia, Jiang et al. 1994](#_ENREF_61), [Prisco, Pagannone et al. 1994](#_ENREF_62), [Stanford and Lacey 1996](#_ENREF_73), [Gobert, Rivet et al. 2000](#_ENREF_30)). DR also excites VTA through postsynaptic 5HT2ARs ([Nocjar, Roth et al. 2002](#_ENREF_56)). LC and VTA excite DR through the noradrenergic receptor 1 (AR1) and D2 receptor (D2R), respectively ([Svensson, Bunney et al. 1975](#_ENREF_74), [Baraban and Aghajanian 1980](#_ENREF_5), [Clement, Gemsa et al. 1992](#_ENREF_17), [Ferre and Artigas 1993](#_ENREF_27), [Martin-Ruiz, Ugedo et al. 2001](#_ENREF_50)). Evidence exists supporting the claim that LC and VTA inhibit one another ([Aghajanian and Bunney 1977](#_ENREF_1), [Elam, Clark et al. 1986](#_ENREF_26), [Grenhoff, North et al. 1995](#_ENREF_32)), while some groups have found that these regions can excite each other ([Deutch, Goldstein et al. 1986](#_ENREF_21), [Grenhoff, Nisell et al. 1993](#_ENREF_31)). Our model contains both excitatory and inhibitory receptors mediating the interactions between these regions. Their respective initial strengths are assigned during the parameter optimization process (see subsection S3). DR and LC also secrete galanin onto one another ([Melander, Hokfelt et al. 1986](#_ENREF_51), [Melander, Hokfelt et al. 1986](#_ENREF_52), [Xu, Zhang et al. 1998](#_ENREF_83), [Lu, Barr et al. 2005](#_ENREF_48)), which binds predominantly to inhibitory galanin receptors (galR1s) in these regions ([Hawes and Picciotto 2004](#_ENREF_37)). There is some binding to excitatory galR2 receptors in the DR as well ([Hawes and Picciotto 2004](#_ENREF_37)). The DR has inhibitory CRF1 and excitatory CRF2 receptors (CRF1R, CRF2R) mediating its responses to CRF innervations from the hypothalamus and the amygdala ([Kirby, Freeman-Daniels et al. 2008](#_ENREF_42), [Spannuth, Hale et al. 2011](#_ENREF_70), [Wood, Zhang et al. 2013](#_ENREF_82)). The LC and VTA both have excitatory CRF1Rs mediating their responses to CRF released from the hypothalamus and the amygdala ([Van Pett, Viau et al. 2000](#_ENREF_79), [Reyes, Valentino et al. 2008](#_ENREF_63), [Wanat, Hopf et al. 2008](#_ENREF_81)).

Each non-monoaminergic unit represents all sources of its corresponding non-monoaminergic transmitter that impinge on the monoaminergic regions from other brain areas. Sources of CRF include amygdala and paraventricular nucleus CRF-producing neurons ([Curtis, Bello et al. 2002](#_ENREF_19), [Hauger, Risbrough et al. 2006](#_ENREF_36), [Rodaros, Caruana et al. 2007](#_ENREF_64)). Widely distributed in the central nervous system (CNS), sources of galanin include the LC, DR, and the hypothalamus, among others ([Melander, Hokfelt et al. 1986](#_ENREF_51)). Sources of glutamate are also widely distributed, and include the cortex and the DR ([Johnson 1994](#_ENREF_39), [Bagley and Moghaddam 1997](#_ENREF_3), [Tassone, Madeo et al. 2011](#_ENREF_76), [Shikanai, Yoshida et al. 2012](#_ENREF_69), [Liu, Zhou et al. 2014](#_ENREF_47)).

Together, the monoaminergic units in the model possess 23 total receptors (of 11 distinct receptor types) to represent their actions on themselves (via autoreceptors), their interactions with each other, and their responses to the non-monoaminergic transmitter units impinging on them. In addition to receptors (monoaminergic units) or generic weights (non-monoaminergic units), each unit also has a bias parameter representing intrinsic influence on its activity. The net input to any unit is then the sum of its inputs from other units, its input from itself, and its own intrinsic bias. For a monoaminergic unit, which has a cognate receptor for each specific transmitter it receives, the amount of released transmitter is multiplied by the strength of the cognate receptor for that transmitter. The net input is the sum of all the receptor-times-transmitter products. For a non-monoaminergic unit, which only has a generic weight for each connection it receives, the activation level of the sending unit is multiplied by the strength of the weight of its connection to the receiving unit. The net input is the sum of the weight-times-activation products (as in a conventional neural network). For any unit, monoaminergic or non-monoaminergic, its intrinsic bias is added to its net input from other units. The activation level of any unit is then computed according to the sigmoidal squashing function as *y* = 1/(1+exp(-*x*)), where *y* is the activation level and *x* is the net input. As such, the activation levels of all units are bounded from 0 to 1.

On each time step, each unit updates its activation level by computing its net input and its resulting output according to the sigmoidal squashing function. Because the amount of released transmitter is equal to the activation level of the releasing unit, the squashing function ensures that no transmitter level falls below zero in the model. The monoaminergic transmitters are the exception, because their levels are reduced by their corresponding transporters by subtracting the numerical value representing the level of the transporter from the amount of released transmitter. Because this could cause the levels to fall below zero, the levels of the monoaminergic transmitters are actively bounded from below at zero (hard limit of zero). Any changes in receptor strength due to drugs are made prior to the unit updates, and any modifications in transmitter levels due to transporters or drugs are made after the updates. The activation levels of all units are set to zero at time step zero, and the units then influence each other’s activation for 150 time steps (see text).

**S3: Details on model parameter optimization**

The parameters of the model were optimized using the genetic algorithm (GA) as implemented in the MATLAB Global Optimization Toolbox. A robust optimization procedure, such as the GA, is appropriate because the model is both nonlinear (sigmoidal squashing function for unit activations) and discontinuous (hard limit of zero for monoaminergic transmitter levels). The 76 real-valued parameters were 23 receptor strengths (on monoaminergic units), 18 connection weights (onto and between non-monoaminergic units), 3 monoaminergic transporters, 29 drug effects (most of the drugs have multiple targets; see Supplementary Table S1), and 6 biases (one for each monoaminergic and non-monoaminergic unit). The 76 parameters form a parameter set that was organized as a vector of 76 elements, where each element is a different parameter. Each run of the GA maintained a population of 100 parameter sets (parameter vectors) and was allowed to “evolve” through simulated mutation and recombination over many generations until the change in fitness between generations was less than a tolerance set to 10^-12^.

We minimized an inverse fitness function (error function) that was based mainly on the difference between the behavior of the monoaminergic units in the model and that of real monoaminergic neurons in their responses to acute administration of various drugs targeting the monoaminergic neurotransmitter systems in rats ([Dong and Blier 2001](#_ENREF_23), [Szabo and Blier 2001](#_ENREF_75), [Chernoloz, El Mansari et al. 2009](#_ENREF_15), [Katz, Guiard et al. 2010](#_ENREF_41), [Ghanbari, El Mansari et al. 2012](#_ENREF_28), [El Mansari, Manta et al. 2015](#_ENREF_25), [Oosterhof, El Mansari et al. 2015](#_ENREF_58)). The rats in these studies were normal, and were not subjected to stressors or other manipulations designed to evoke a depressive phenotype (ibid). The drugs and drug combinations included in the error function constitute the set of drugs that target the monoaminergic neurotransmitter systems studied by Pierre Blier and his lab, with the exceptions of GBR12909 (GBR) and Pramipexole (PPX).

GBR is a high-affinity DA reuptake inhibitor, with 500-fold higher affinity for the dopamine transporter (DAT) than for the other monoaminergic transporters ([Andersen 1989](#_ENREF_2)). It does not have significant affinity for any other receptors ([TOCRIS](#_ENREF_78) , [Andersen 1989](#_ENREF_2), [Rothman, Mele et al. 1991](#_ENREF_66)). The Blier group found a 26% decrease in VTA DA neuron firing in response to acute GBR, with no change in the firing parameters of LC NE neurons or DR 5HT neurons ([Katz, Guiard et al. 2010](#_ENREF_41)). This is inconsistent with the behavior of the other reuptake blockers, Escitalopram, Nomifensine, and Reboxetine, which all produce changes in neuronal firing rate in more than one monoaminergic region ([Katz, Guiard et al. 2010](#_ENREF_41), [Chernoloz, El Mansari et al. 2012](#_ENREF_16)). During GA searches, the strength of GBR was repeatedly pushed to zero by the algorithm. Due to inconsistency of GBR data with the behavior of the other reuptake inhibitors, as well as consistent GA assignment of zero strength for GBR in the model, data obtained from GBR experiments was removed from the error function and was not considered further in model analysis.

PPX primarily targets D3 receptors (D3R) ([Mierau, Schneider et al. 1995](#_ENREF_53)). PPX has been found to be 8 times more potent as a D3 receptor agonist than as a D2 receptor agonist through functional measurements of receptor activation ([Mierau, Schneider et al. 1995](#_ENREF_53)). The dissociation constant (Ki) of PPX is 0.5 nM at D3R and 3.9 nM at D2R, demonstrating that the binding affinity of PPX at D3R is almost 8 times higher than that of PPX at D2R ([Newman-Tancredi, Cussac et al. 2002](#_ENREF_54)). D3Rs are mainly expressed in the nucleus accumbens, hypothalamus, and prefrontal cortex, but are not present in significant levels in the monoaminergic regions ([Levesque, Diaz et al. 1992](#_ENREF_45)). In this initial model, receptors are represented explicitly only on monoaminergic units, so D3Rs are not represented. PPX data was not included in the error function nor was it considered in model analysis because its primary target, D3R, is not represented in the model.

Interpretations of data by different groups ascribe to Bupropion a mechanism of action that elevates NE and DA through either a releasing ([Dong and Blier 2001](#_ENREF_23)) or reuptake blocking (NDRI) mechanism ([Cooper, Wang et al. 1994](#_ENREF_18)). The Blier lab adheres to the releasing mechanism interpretation, which is the mechanism that is implemented in the model. To study the effects of Quetiapine, the Blier lab used a compound combination they called Human Quetiapine. Because humans metabolize Quetiapine to N-Desalkyl quetiapine (Nquet) but rodents do not, rodents were given a mixture of Nquet and Quetiapine in the ratio present in humans during the metabolism of quetiapine to produce what the Blier group calls “human Quetiapine” ([Chernoloz, El Mansari et al. 2012](#_ENREF_16)). We simulated the effects of “human Quetiapine” in the model by giving Quetiapine all the targets of both Quetiapine and Nquet (see Supplementary Table S1).

The error function that was minimized by the GA computed a modified root mean square (RMS) error. The heart of the error function is the differences between the observed percentage changes and the percentage changes in the average activation levels of the monoaminergic units in the model due to acute drug administration, as described above. Acute drug data were obtained from the experimental findings of the Blier group. Through reduction of error, the GA had to optimize several criteria in addition to the agreement in the percentage changes in monoaminergic activation levels due to acute drugs. The baseline (no-drug) activity levels of the 6 units were required to be near 0.5, which is the midpoint of the unit activity range as governed by the squashing function. This gave the units maximal freedom to increase or decrease their activations with drug administrations or receptor strength adjustments. Monoaminergic baseline (no-drug) neurotransmitter levels had to be 0.1 or greater. This ensured that pre-drug transmitter levels were high enough for all drugs to have a substantial effect. The rise in 5HT following acute Escitalopram had to be 0.2 or greater, and the rise in NE following acute Reboxetine had to be 0.2 or greater. This ensured that levels of 5HT and NE due to acute blockade of their respective transporters agreed with experimental findings ([Kreiss and Lucki 1995](#_ENREF_44), [Malagie, Trillat et al. 1995](#_ENREF_49), [Bymaster, Zhang et al. 2002](#_ENREF_12), [Page, Brown et al. 2003](#_ENREF_60), [Romero, Celada et al. 2003](#_ENREF_65)).

For programming convenience, parameters whose values could be positive or negative (ie, strengths of receptors, transporters, and drugs) were set as absolute values and their signs were fixed in program code. Other parameters (ie, generic weights and unit biases) were signed by the optimization procedure. Lower and upper bounds for the parameter values in the GA were set by hand. Lower and upper bounds for the generic weights of the connections onto non-monoaminergic transmitter units, and for all unit biases, were set at -10 and +10. Lower and upper autoreceptor bounds were set at 3 and 10, respectively. The GA-determined levels for all autoreceptors (DR 5HT1A, LC AR2, and VTA D2R) were near 3 in almost all 200 parameterizations and in all of the “ten-best” parameterizations (see below). All other receptor bounds were set at 1 and 10. Transporter bounds were set at 0.3 and 0.9. Bounds for the strengths of the drugs were set at 0 and 1. The bounds on the strengths of drugs provided consistency in effects for drugs of different classes in the model.

All receptors contributed a component to net input equal to the product of their strength and the level of their cognate transmitter, regardless of whether or not a drug that affects them was present. Agonist drugs add a contribution from a receptor equal to the product of the receptor strength and the drug strength. Antagonist drugs subtract a component equal to the product of the receptor strength, the drug strength, and the level of the cognate transmitter. Because transmitters and drugs are bounded between 0 and 1, this scheme ensures that an agonist drug can increase the receptor contribution at most by one full receptor strength, while an antagonist can decrease the receptor contribution at most to zero. Transporter blocking drugs worked similarly, reducing the level of a transporter by an amount equal to the product of the transporter strength and the drug strength. Therefore, a blocker could reduce the strength of a transporter at most to zero. Transmitter releasing drugs add a contribution of neurotransmitter to the unit activation level equal to the product of the amount of released transmitter (which is equal to the unit activation) and the drug strength. Because the releasing drug strengths (like all drug strengths) are bounded between 0 and 1, this ensures that a releasing drug can at most double the amount of released transmitter.

In the error function, differences in the no-drug monoaminergic unit activations from 0.5, and differences between the observed and simulated monoaminergic activation percentage changes due to acute drug, were expressed as actual RMS differences (ie, RMS errors). However, each no-drug monoaminergic transmitter level below 0.1, and each 5HT or NE level below 0.2 with acute Escitalopram or Reboxetine, respectively, conditionally contributed a value of 100 to the error term for the offending parameter set. Because discrepancies between desired and simulated transmitter levels were expressed as conditionals, our error function computed a modified RMS error measure.

The 10 lowest-error parameter sets of the 200 GA searches were separated out for further consideration. For all of these “ten best” parameterizations, the transmitter levels achieved their required levels. Therefore, the conditional contributions to the error functions were zero, leaving only the actual RMS differences in desired and actual monoaminergic unit activities. Note that the purpose of the GA optimization was to set baseline parameter values according to the data on the acute effects of drugs. Data on chronic drug effects, which involve adjustments in baseline receptor values, were therefore not represented in the fitness function.

**S4: Comparison with other models of the monoaminergic neurotransmitter systems**

A mathematical model of serotonin synthesis, release, and reuptake in axon terminals was developed by one group to better understand the effects of autoreceptor activation on DA and 5HT neuron firing patterns ([Best, Nijhout et al. 2010](#_ENREF_7), [Best, Reed et al. 2010](#_ENREF_9), [Best, Nijhout et al. 2011](#_ENREF_8)). The same group used mathematical models of DR neurons with normal and low vesicular 5HT to study the adaptation of DR neurons to chronic SSRI. Through the use of differential equations that represent the concentrations and activity levels of different proteins involved in 5HT metabolism and release, the authors demonstrated that acute doses of SSRIs do not bring the burst firing of DR neurons back to normal, but chronic use of SSRIs do return the burst response to normative levels. Although many models of DA-producing neurons exist, one group notably produced a neuro-computational model of VTA afferents to better understand temporal learning rules, designing an architecture that is similar to ours in that it includes multiple units representing non-monoaminergic neurons that interact with a unit representing a monoaminergic (specifically a VTA) neuron ([Vitay and Hamker 2014](#_ENREF_80)).

Our model has features in common with these models, namely that they all represent neuron activity levels and average concentrations of transmitters. Our computational model can be distinguished from the others in that it is the first of its kind to represent interactions between all three monoaminergic brain regions and regions producing non-monoaminergic transmitters. Among the potentially important factors left out of our initial model are presynaptic receptors, but they can be included in subsequent model versions. In the initial model, only the activity levels of the monoaminergic units are included in the activation error term. The non-monoaminergic transmitter units (tCRF, Tgal, and Tglu) are not included in the activation error calculation because they group together neurons from various brain regions that most likely do not all undergo drug-dependent changes of the same magnitude and direction. The non-monoaminergic units could be differentiated into units representing discrete brain regions in subsequent, expanded versions of the model. Other neurotransmitter, neuropeptide, and neurohormone systems, and their associated neural source and target regions, could also be added.

# Supplementary Figures and Tables

**Supplementary Table S1**

**Summary of drugs and drug combinations considered in the analysis**

Each row contains the name of a drug, its class, current uses, its effects as represented in the model, and supporting references. These drugs and drug combinations were used in the experiments by the Blier lab that determined the percent changes from baseline in the firing rates of neurons in the monoaminergic nuclei after acute (2-day) and chronic (14-day) drug administration. The drug types include transporter blockers, receptor agonists, receptor antagonists, and neurotransmitter releasers. The strengths of each of these drugs at their targets were determined using the GA implemented in MATLAB.

| **Drug name** | **Drug class** | **Drug uses** | **Drug effects at targets** | **References** |
| --- | --- | --- | --- | --- |
| Escitalopram | 2^nd^ generation antidepressant | Depression | 5HT transporter blocker | ([Sanchez, Bergqvist et al. 2003](#_ENREF_67), [Chen, Larsen et al. 2005](#_ENREF_14)) |
| Nomifensine | 2^nd^ generation antidepressant | Depression | DA, NE and 5HT transporter blocker | ([Brogden, Heel et al. 1979](#_ENREF_11), [Tatsumi, Groshan et al. 1997](#_ENREF_77)) |
| Reboxetine | 2^nd^ generation antidepressant | Depression, panic disorder, attention deficit hyperactivity disorder (ADHD) | NE transporter blocker | ([Page, Brown et al. 2003](#_ENREF_60), [Hajos, Fleishaker et al. 2004](#_ENREF_34)) |
| Trazodone | Tetracyclic antidepressant | Depression, post-traumatic stress disorder (PTSD), insomnia | 5HT transporter blocker, agonist at 5HT1AR, antagonist at 5HT2AR, 5HT2CR, AR1, AR2, and D2R | ([Cusack, Nelson et al. 1994](#_ENREF_20), [Nierenberg, Adler et al. 1994](#_ENREF_55), [Krege, Goepel et al. 2000](#_ENREF_43), [Balsara, Jadhav et al. 2005](#_ENREF_4), [Odagaki, Toyoshima et al. 2005](#_ENREF_57), [Stahl 2009](#_ENREF_71), [Stahl 2009](#_ENREF_72)) |
| Asenapine | Atypical antipsychotic | Bipolar disorder, schizophrenia | Agonist at 5HT1AR, antagonist at 5HT2AR, AR2, and D2R | ([Ghanbari, El Mansari et al. 2009](#_ENREF_29), [Bjorkholm, Franberg et al. 2015](#_ENREF_10)) |
| Aripiprazole | Atypical antipsychotic | Bipolar disorder, schizophrenia | Agonist at 5HT1AR and D2R, antagonist at 5HT2AR and 5HT2CR | ([Shapiro, Renock et al. 2003](#_ENREF_68), [Li, Ichikawa et al. 2004](#_ENREF_46), [Han, Wang et al. 2013](#_ENREF_35)) |
| Bupropion | Atypical antidepressant | Depression, seasonal affective disorder | Releaser of NE and DA | ([Cooper, Wang et al. 1994](#_ENREF_18), [Cusack, Nelson et al. 1994](#_ENREF_20), [Dong and Blier 2001](#_ENREF_23), [El Mansari, Ghanbari et al. 2008](#_ENREF_24)) |
| Quetiapine | Atypical antipsychotic | Bipolar disorder, schizophrenia, depression | NE transporter blocker, agonist at 5HT1AR, antagonist at 5HT2AR, AR1, AR2, D1R, and D2R | ([DeVane and Nemeroff 2001](#_ENREF_22), [Jensen, Rodriguiz et al. 2008](#_ENREF_38), [Han, Wang et al. 2013](#_ENREF_35)) |

**Supplementary Figure S1**

**Desensitization of the 5HT1A autoreceptor on the DR unit**

a) Neuroadaptation to chronic Escitalopram decreases activation error in the model, thereby bringing the activations of the monoaminergic units back towards their normative levels. b) In this single-sequence adaptation run, only the 5HT1A receptor on the DR unit (the 5HT1A autoreceptor) was allowed to adapt. It could adjust its strength up or down by 1 on each of five adjustments, but the adjustment is retained only if it results in a decrease in activation error. The receptor decrements to 0 over adjustment steps 2, 3, and 4. No change occurs on adjustment step 1 because the randomly chosen adjustment was an increment, but that adjustment was rejected because it did not decrease activation error. Similarly, no change occurs on the last step because no further error-reducing adjustment can be made.

**
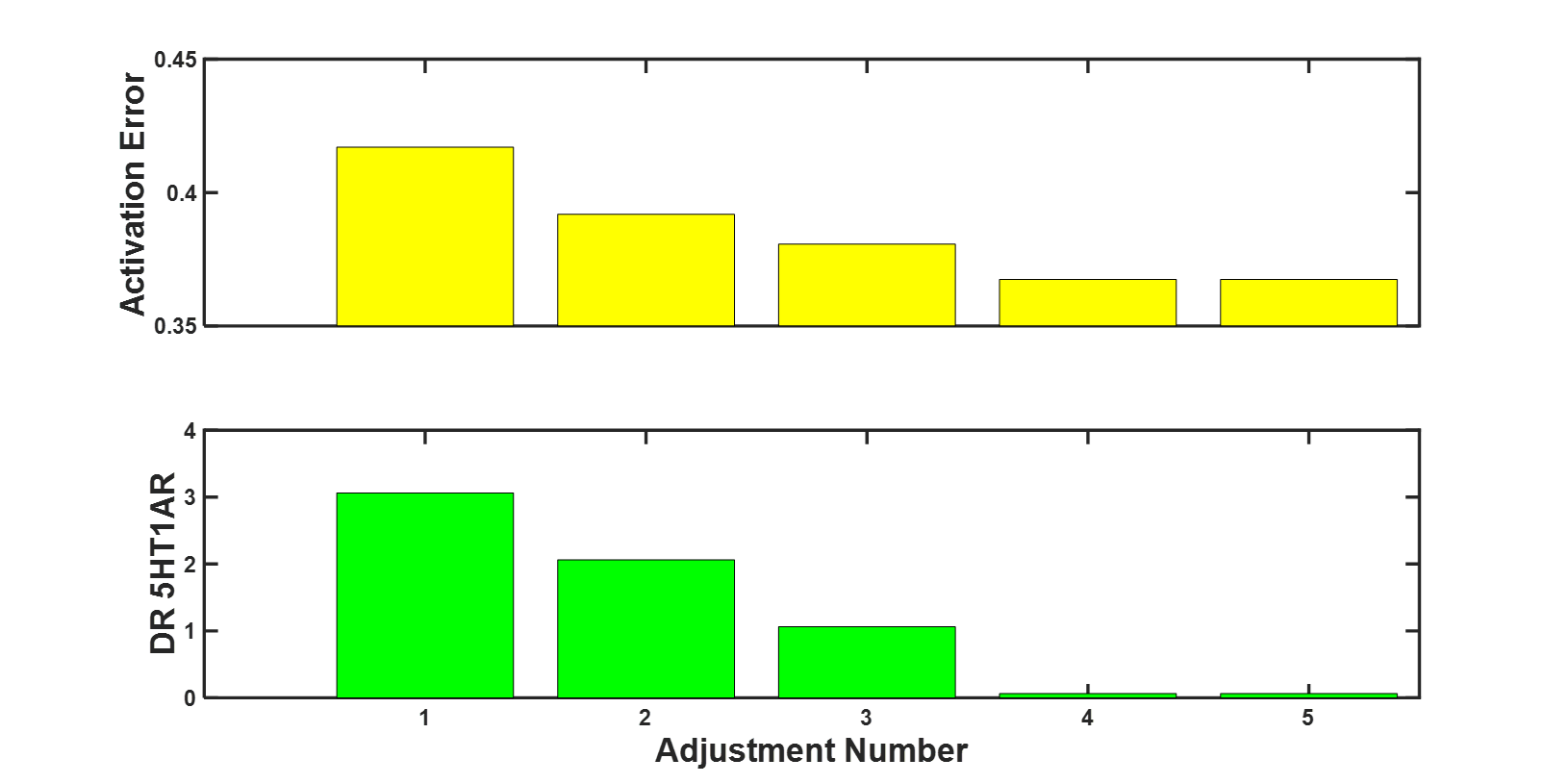
**

**Supplementary Figure S2**

**Frequency of MATLAB Receptor Adjustment Sequence Lengths**

The MATLAB version of the model was used for making receptor strength adjustments along single sequences in which adjustment of a randomly chosen receptor was retained only if it resulted in a homeostatic reduction in activation error. Here, 1,000,000 randomly-ordered sequences of strictly error-reducing adjustments were allowed to continue until the model achieved complete adaptation, in which further adjustments no longer reduced activation error with chronic Escitalopram. The histogram plots the number of sequences achieving complete adaptation as a function of the number of adjustments. The mean number of adjustments is 7.9, the mode is 7, the median is 8, and the range is from 2 to 26.

**Supplementary Figure S3**

**Heat map of 1000 terminal receptor strength configurations with chronic Escitalopram**

The MATLAB version of the model was used to make 1000 randomly-ordered, strictly error-reducing sequences of adjustments until further adjustments no longer reduced the activation error with chronic Escitalopram. This approach produced 1000 completely adapted receptor state configurations, which we plotted using a heat map. Each of 1000 rows is a vector of final receptor values for all 11 adjustable receptors. The 1000 completely adapted configurations were ordered by Euclidian distance from a reference vector of all zeros, which is shown in row 0. The 11 adjustable receptors can adjust by 1 to any level between 0 and 10 for a total of 11^11^ or more than 285 billion possible receptor strength configurations. This very small sample of 1000 completely adapted configurations reveals considerable heterogeneity, and clearly shows that there are very many configurations that are completely adapted in the sense that further receptor strength adjustment will not further decrease activation error.


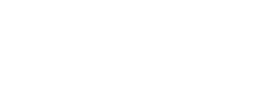


**References**

Aghajanian, G. K. and B. S. Bunney (1977). "Dopamine"autoreceptors": pharmacological characterization by microiontophoretic single cell recording studies." Naunyn Schmiedebergs Arch Pharmacol **297**(1): 1-7.

Andersen, P. H. (1989). "The dopamine inhibitor GBR 12909: selectivity and molecular mechanism of action." Eur J Pharmacol **166**(3): 493-504.

Bagley, J. and B. Moghaddam (1997). "Temporal dynamics of glutamate efflux in the prefrontal cortex and in the hippocampus following repeated stress: effects of pretreatment with saline or diazepam." Neuroscience **77**(1): 65-73.

Balsara, J. J., S. A. Jadhav, R. K. Gaonkar, R. V. Gaikwad and J. H. Jadhav (2005). "Effects of the antidepressant trazodone, a 5-HT 2A/2C receptor antagonist, on dopamine-dependent behaviors in rats." Psychopharmacology (Berl) **179**(3): 597-605.

Baraban, J. M. and G. K. Aghajanian (1980). "Suppression of firing activity of 5-HT neurons in the dorsal raphe by alpha-adrenoceptor antagonists." Neuropharmacology **19**(4): 355-363.

Beckstead, R. M., V. B. Domesick and W. J. Nauta (1979). "Efferent connections of the substantia nigra and ventral tegmental area in the rat." Brain Res **175**(2): 191-217.

Best, J., H. F. Nijhout and M. Reed (2010). "Serotonin synthesis, release and reuptake in terminals: a mathematical model." Theor Biol Med Model **7**: 34.

Best, J., H. F. Nijhout and M. Reed (2011). "Bursts and the efficacy of selective serotonin reuptake inhibitors." Pharmacopsychiatry **44 Suppl 1**: S76-83.

Best, J., M. Reed and H. F. Nijhout (2010). "Models of dopaminergic and serotonergic signaling." Pharmacopsychiatry **43 Suppl 1**: S61-66.

Bjorkholm, C., O. Franberg, A. Malmerfelt, M. M. Marcus, A. Konradsson-Geuken, B. Schilstrom, K. Jardemark and T. H. Svensson (2015). "Adjunctive treatment with asenapine augments the escitalopram-induced effects on monoaminergic outflow and glutamatergic neurotransmission in the medial prefrontal cortex of the rat." Int J Neuropsychopharmacol **18**(3).

Brogden, R. N., R. C. Heel, T. M. Speight and G. S. Avery (1979). "Nomifensine: A review of its pharmacological properties and therapeutic efficacy in depressive illness." Drugs **18**(1): 1-24.

Bymaster, F. P., W. Zhang, P. A. Carter, J. Shaw, E. Chernet, L. Phebus, D. T. Wong and K. W. Perry (2002). "Fluoxetine, but not other selective serotonin uptake inhibitors, increases norepinephrine and dopamine extracellular levels in prefrontal cortex." Psychopharmacology (Berl) **160**(4): 353-361.

Can, A., D. T. Dao, M. Arad, C. E. Terrillion, S. C. Piantadosi and T. D. Gould (2012). "The mouse forced swim test." J Vis Exp(59): e3638.

Chen, F., M. B. Larsen, C. Sanchez and O. Wiborg (2005). "The S-enantiomer of R,S-citalopram, increases inhibitor binding to the human serotonin transporter by an allosteric mechanism. Comparison with other serotonin transporter inhibitors." Eur Neuropsychopharmacol **15**(2): 193-198.

Chernoloz, O., M. El Mansari and P. Blier (2009). "Electrophysiological studies in the rat brain on the basis for aripiprazole augmentation of antidepressants in major depressive disorder." Psychopharmacology (Berl) **206**(2): 335-344.

Chernoloz, O., M. El Mansari and P. Blier (2012). "Effects of sustained administration of quetiapine alone and in combination with a serotonin reuptake inhibitor on norepinephrine and serotonin transmission." Neuropsychopharmacology **37**(7): 1717-1728.

Clement, H. W., D. Gemsa and W. Wesemann (1992). "The effect of adrenergic drugs on serotonin metabolism in the nucleus raphe dorsalis of the rat, studied by in vivo voltammetry." Eur J Pharmacol **217**(1): 43-48.

Cooper, B. R., C. M. Wang, R. F. Cox, R. Norton, V. Shea and R. M. Ferris (1994). "Evidence that the acute behavioral and electrophysiological effects of bupropion (Wellbutrin) are mediated by a noradrenergic mechanism." Neuropsychopharmacology **11**(2): 133-141.

Curtis, A. L., N. T. Bello, K. R. Connolly and R. J. Valentino (2002). "Corticotropin-releasing factor neurones of the central nucleus of the amygdala mediate locus coeruleus activation by cardiovascular stress." J Neuroendocrinol **14**(8): 667-682.

Cusack, B., A. Nelson and E. Richelson (1994). "Binding of antidepressants to human brain receptors: focus on newer generation compounds." Psychopharmacology (Berl) **114**(4): 559-565.

Deutch, A. Y., M. Goldstein and R. H. Roth (1986). "Activation of the locus coeruleus induced by selective stimulation of the ventral tegmental area." Brain Res **363**(2): 307-314.

DeVane, C. L. and C. B. Nemeroff (2001). "Clinical pharmacokinetics of quetiapine: an atypical antipsychotic." Clin Pharmacokinet **40**(7): 509-522.

Dong, J. and P. Blier (2001). "Modification of norepinephrine and serotonin, but not dopamine, neuron firing by sustained bupropion treatment." Psychopharmacology (Berl) **155**(1): 52-57.

El Mansari, M., R. Ghanbari, S. Janssen and P. Blier (2008). "Sustained administration of bupropion alters the neuronal activity of serotonin, norepinephrine but not dopamine neurons in the rat brain." Neuropharmacology **55**(7): 1191-1198.

El Mansari, M., S. Manta, C. Oosterhof, K. S. El Iskandrani, F. Chenu, S. Shim and P. Blier (2015). "Restoration of serotonin neuronal firing following long-term administration of bupropion but not paroxetine in olfactory bulbectomized rats." Int J Neuropsychopharmacol **18**(4).

Elam, M., D. Clark and T. H. Svensson (1986). "Electrophysiological effects of the enantiomers of 3-PPP on neurons in the locus coeruleus of the rat." Neuropharmacology **25**(9): 1003-1008.

Ferre, S. and F. Artigas (1993). "Dopamine D2 receptor-mediated regulation of serotonin extracellular concentration in the dorsal raphe nucleus of freely moving rats." J Neurochem **61**(2): 772-775.

Ghanbari, R., M. El Mansari and P. Blier (2012). "Electrophysiological impact of trazodone on the dopamine and norepinephrine systems in the rat brain." Eur Neuropsychopharmacol **22**(7): 518-526.

Ghanbari, R., M. El Mansari, M. Shahid and P. Blier (2009). "Electrophysiological characterization of the effects of asenapine at 5-HT(1A), 5-HT(2A), alpha(2)-adrenergic and D(2) receptors in the rat brain." Eur Neuropsychopharmacol **19**(3): 177-187.

Gobert, A., J. M. Rivet, F. Lejeune, A. Newman-Tancredi, A. Adhumeau-Auclair, J. P. Nicolas, L. Cistarelli, C. Melon and M. J. Millan (2000). "Serotonin(2C) receptors tonically suppress the activity of mesocortical dopaminergic and adrenergic, but not serotonergic, pathways: a combined dialysis and electrophysiological analysis in the rat." Synapse **36**(3): 205-221.

Grenhoff, J., M. Nisell, S. Ferre, G. Aston-Jones and T. H. Svensson (1993). "Noradrenergic modulation of midbrain dopamine cell firing elicited by stimulation of the locus coeruleus in the rat." J Neural Transm Gen Sect **93**(1): 11-25.

Grenhoff, J., R. A. North and S. W. Johnson (1995). "Alpha 1-adrenergic effects on dopamine neurons recorded intracellularly in the rat midbrain slice." Eur J Neurosci **7**(8): 1707-1713.

Guiard, B. P., M. El Mansari and P. Blier (2008). "Cross-talk between dopaminergic and noradrenergic systems in the rat ventral tegmental area, locus ceruleus, and dorsal hippocampus." Mol Pharmacol **74**(5): 1463-1475.

Hajos, M., J. C. Fleishaker, J. K. Filipiak-Reisner, M. T. Brown and E. H. F. Wong (2004). "The selective norepinephrine reuptake inhibitor antidepressant reboxetine: Pharmacological and clinical profile." Cns Drug Reviews **10**(1): 23-44.

Han, C., S. M. Wang, M. Kato, S. J. Lee, A. A. Patkar, P. S. Masand and C. U. Pae (2013). "Second-generation antipsychotics in the treatment of major depressive disorder: current evidence." Expert Rev Neurother **13**(7): 851-870.

Hauger, R. L., V. Risbrough, O. Brauns and F. M. Dautzenberg (2006). "Corticotropin releasing factor (CRF) receptor signaling in the central nervous system: new molecular targets." CNS Neurol Disord Drug Targets **5**(4): 453-479.

Hawes, J. J. and M. R. Picciotto (2004). "Characterization of GalR1, GalR2, and GalR3 immunoreactivity in catecholaminergic nuclei of the mouse brain." J Comp Neurol **479**(4): 410-423.

Jensen, N. H., R. M. Rodriguiz, M. G. Caron, W. C. Wetsel, R. B. Rothman and B. L. Roth (2008). "N-desalkylquetiapine, a potent norepinephrine reuptake inhibitor and partial 5-HT1A agonist, as a putative mediator of quetiapine's antidepressant activity." Neuropsychopharmacology **33**(10): 2303-2312.

Johnson, M. D. (1994). "Synaptic glutamate release by postnatal rat serotonergic neurons in microculture." Neuron **12**(2): 433-442.

Kalen, P., G. Skagerberg and O. Lindvall (1988). "Projections from the ventral tegmental area and mesencephalic raphe to the dorsal raphe nucleus in the rat. Evidence for a minor dopaminergic component." Exp Brain Res **73**(1): 69-77.

Katz, N. S., B. P. Guiard, M. El Mansari and P. Blier (2010). "Effects of acute and sustained administration of the catecholamine reuptake inhibitor nomifensine on the firing activity of monoaminergic neurons." J Psychopharmacol **24**(8): 1223-1235.

Kirby, L. G., E. Freeman-Daniels, J. C. Lemos, J. D. Nunan, C. Lamy, A. Akanwa and S. G. Beck (2008). "Corticotropin-releasing factor increases GABA synaptic activity and induces inward current in 5-hydroxytryptamine dorsal raphe neurons." J Neurosci **28**(48): 12927-12937.

Krege, S., M. Goepel, H. Sperling and M. C. Michel (2000). "Affinity of trazodone for human penile alpha1- andalpha2-adrenoceptors." BJU Int **85**(7): 959-961.

Kreiss, D. S. and I. Lucki (1995). "Effects of acute and repeated administration of antidepressant drugs on extracellular levels of 5-hydroxytryptamine measured in vivo." J Pharmacol Exp Ther **274**(2): 866-876.

Levesque, D., J. Diaz, C. Pilon, M. P. Martres, B. Giros, E. Souil, D. Schott, J. L. Morgat, J. C. Schwartz and P. Sokoloff (1992). "Identification, characterization, and localization of the dopamine D3 receptor in rat brain using 7-[3H]hydroxy-N,N-di-n-propyl-2-aminotetralin." Proc Natl Acad Sci U S A **89**(17): 8155-8159.

Li, Z., J. Ichikawa, J. Dai and H. Y. Meltzer (2004). "Aripiprazole, a novel antipsychotic drug, preferentially increases dopamine release in the prefrontal cortex and hippocampus in rat brain." Eur J Pharmacol **493**(1-3): 75-83.

Liu, Z., J. Zhou, Y. Li, F. Hu, Y. Lu, M. Ma, Q. Feng, J. E. Zhang, D. Wang, J. Zeng, J. Bao, J. Y. Kim, Z. F. Chen, S. El Mestikawy and M. Luo (2014). "Dorsal raphe neurons signal reward through 5-HT and glutamate." Neuron **81**(6): 1360-1374.

Lu, X., A. M. Barr, J. W. Kinney, P. Sanna, B. Conti, M. M. Behrens and T. Bartfai (2005). "A role for galanin in antidepressant actions with a focus on the dorsal raphe nucleus." Proc Natl Acad Sci U S A **102**(3): 874-879.

Malagie, I., A. C. Trillat, C. Jacquot and A. M. Gardier (1995). "Effects of acute fluoxetine on extracellular serotonin levels in the raphe: an in vivo microdialysis study." Eur J Pharmacol **286**(2): 213-217.

Martin-Ruiz, R., L. Ugedo, M. A. Honrubia, G. Mengod and F. Artigas (2001). "Control of serotonergic neurons in rat brain by dopaminergic receptors outside the dorsal raphe nucleus." J Neurochem **77**(3): 762-775.

Melander, T., T. Hokfelt and A. Rokaeus (1986). "Distribution of Galanin-Like Immunoreactivity in the Rat Central-Nervous-System." Journal of Comparative Neurology **248**(4): 475-517.

Melander, T., T. Hokfelt, A. Rokaeus, A. C. Cuello, W. H. Oertel, A. Verhofstad and M. Goldstein (1986). "Coexistence of galanin-like immunoreactivity with catecholamines, 5-hydroxytryptamine, GABA and neuropeptides in the rat CNS." J Neurosci **6**(12): 3640-3654.

Mierau, J., F. J. Schneider, H. A. Ensinger, C. L. Chio, M. E. Lajiness and R. M. Huff (1995). "Pramipexole binding and activation of cloned and expressed dopamine D2, D3 and D4 receptors." Eur J Pharmacol **290**(1): 29-36.

Newman-Tancredi, A., D. Cussac, Y. Quentric, M. Touzard, L. Verriele, N. Carpentier and M. J. Millan (2002). "Differential actions of antiparkinson agents at multiple classes of monoaminergic receptor. III. Agonist and antagonist properties at serotonin, 5-HT(1) and 5-HT(2), receptor subtypes." J Pharmacol Exp Ther **303**(2): 815-822.

Nierenberg, A. A., L. A. Adler, E. Peselow, G. Zornberg and M. Rosenthal (1994). "Trazodone for antidepressant-associated insomnia." Am J Psychiatry **151**(7): 1069-1072.

Nocjar, C., B. L. Roth and E. A. Pehek (2002). "Localization of 5-HT(2A) receptors on dopamine cells in subnuclei of the midbrain A10 cell group." Neuroscience **111**(1): 163-176.

Odagaki, Y., R. Toyoshima and T. Yamauchi (2005). "Trazodone and its active metabolite m-chlorophenylpiperazine as partial agonists at 5-HT1A receptors assessed by [35S]GTPgammaS binding." J Psychopharmacol **19**(3): 235-241.

Oosterhof, C. A., M. El Mansari and P. Blier (2015). "Asenapine alters the activity of monoaminergic systems following its subacute and long-term administration: An in vivo electrophysiological characterization." Eur Neuropsychopharmacol.

Ornstein, K., H. Milon, A. McRae-Degueurce, C. Alvarez, B. Berger and H. P. Wurzner (1987). "Biochemical and radioautographic evidence for dopaminergic afferents of the locus coeruleus originating in the ventral tegmental area." J Neural Transm **70**(3-4): 183-191.

Page, M. E., K. Brown and I. Lucki (2003). "Simultaneous analyses of the neurochemical and behavioral effects of the norepinephrine reuptake inhibitor reboxetine in a rat model of antidepressant action." Psychopharmacology (Berl) **165**(2): 194-201.

Pessia, M., Z. G. Jiang, R. A. North and S. W. Johnson (1994). "Actions of 5-hydroxytryptamine on ventral tegmental area neurons of the rat in vitro." Brain Res **654**(2): 324-330.

Prisco, S., S. Pagannone and E. Esposito (1994). "Serotonin-dopamine interaction in the rat ventral tegmental area: an electrophysiological study in vivo." J Pharmacol Exp Ther **271**(1): 83-90.

Reyes, B. A., R. J. Valentino and E. J. Van Bockstaele (2008). "Stress-induced intracellular trafficking of corticotropin-releasing factor receptors in rat locus coeruleus neurons." Endocrinology **149**(1): 122-130.

Rodaros, D., D. A. Caruana, S. Amir and J. Stewart (2007). "Corticotropin-releasing factor projections from limbic forebrain and paraventricular nucleus of the hypothalamus to the region of the ventral tegmental area." Neuroscience **150**(1): 8-13.

Romero, L., P. Celada, R. Martin-Ruiz, L. Diaz-Mataix, M. Mourelle, J. Delgadillo, I. Hervas and F. Artigas (2003). "Modulation of serotonergic function in rat brain by VN2222, a serotonin reuptake inhibitor and 5-HT1A receptor agonist." Neuropsychopharmacology **28**(3): 445-456.

Rothman, R. B., A. Mele, A. A. Reid, H. C. Akunne, N. Greig, A. Thurkauf, B. R. de Costa, K. C. Rice and A. Pert (1991). "GBR12909 antagonizes the ability of cocaine to elevate extracellular levels of dopamine." Pharmacol Biochem Behav **40**(2): 387-397.

Sanchez, C., P. B. Bergqvist, L. T. Brennum, S. Gupta, S. Hogg, A. Larsen and O. Wiborg (2003). "Escitalopram, the S-(+)-enantiomer of citalopram, is a selective serotonin reuptake inhibitor with potent effects in animal models predictive of antidepressant and anxiolytic activities." Psychopharmacology (Berl) **167**(4): 353-362.

Shapiro, D. A., S. Renock, E. Arrington, L. A. Chiodo, L. X. Liu, D. R. Sibley, B. L. Roth and R. Mailman (2003). "Aripiprazole, a novel atypical antipsychotic drug with a unique and robust pharmacology." Neuropsychopharmacology **28**(8): 1400-1411.

Shikanai, H., T. Yoshida, K. Konno, M. Yamasaki, T. Izumi, Y. Ohmura, M. Watanabe and M. Yoshioka (2012). "Distinct neurochemical and functional properties of GAD67-containing 5-HT neurons in the rat dorsal raphe nucleus." J Neurosci **32**(41): 14415-14426.

Spannuth, B. M., M. W. Hale, A. K. Evans, J. L. Lukkes, S. Campeau and C. A. Lowry (2011). "Investigation of a central nucleus of the amygdala/dorsal raphe nucleus serotonergic circuit implicated in fear-potentiated startle." Neuroscience **179**: 104-119.

Stahl, S. M. (2009). "Mechanism of action of trazodone: a multifunctional drug." CNS Spectr **14**(10): 536-546.

Stahl, S. M. (2009). Stahls Essential Psychopharmacology Prescribers Guide. New York, NY, Cambridge University Press.

Stanford, I. M. and M. G. Lacey (1996). "Differential actions of serotonin, mediated by 5-HT1B and 5-HT2C receptors, on GABA-mediated synaptic input to rat substantia nigra pars reticulata neurons in vitro." J Neurosci **16**(23): 7566-7573.

Svensson, T. H., B. S. Bunney and G. K. Aghajanian (1975). "Inhibition of both noradrenergic and serotonergic neurons in brain by the alpha-adrenergic agonist clonidine." Brain Res **92**(2): 291-306.

Szabo, S. T. and P. Blier (2001). "Effect of the selective noradrenergic reuptake inhibitor reboxetine on the firing activity of noradrenaline and serotonin neurons." Eur J Neurosci **13**(11): 2077-2087.

Tassone, A., G. Madeo, T. Schirinzi, D. Vita, F. Puglisi, G. Ponterio, F. Borsini, A. Pisani and P. Bonsi (2011). "Activation of 5-HT6 receptors inhibits corticostriatal glutamatergic transmission." Neuropharmacology **61**(4): 632-637.

Tatsumi, M., K. Groshan, R. D. Blakely and E. Richelson (1997). "Pharmacological profile of antidepressants and related compounds at human monoamine transporters." Eur J Pharmacol **340**(2-3): 249-258.

TOCRIS GBR 12909 dihydrochloride. T. a. b. brand.

Van Pett, K., V. Viau, J. C. Bittencourt, R. K. Chan, H. Y. Li, C. Arias, G. S. Prins, M. Perrin, W. Vale and P. E. Sawchenko (2000). "Distribution of mRNAs encoding CRF receptors in brain and pituitary of rat and mouse." J Comp Neurol **428**(2): 191-212.

Vitay, J. and F. H. Hamker (2014). "Timing and expectation of reward: a neuro-computational model of the afferents to the ventral tegmental area." Front Neurorobot **8**: 4.

Wanat, M. J., F. W. Hopf, G. D. Stuber, P. E. Phillips and A. Bonci (2008). "Corticotropin-releasing factor increases mouse ventral tegmental area dopamine neuron firing through a protein kinase C-dependent enhancement of Ih." J Physiol **586**(8): 2157-2170.

Wood, S. K., X. Y. Zhang, B. A. Reyes, C. S. Lee, E. J. Van Bockstaele and R. J. Valentino (2013). "Cellular adaptations of dorsal raphe serotonin neurons associated with the development of active coping in response to social stress." Biol Psychiatry **73**(11): 1087-1094.

Xu, Z. Q., X. Zhang, V. A. Pieribone, S. Grillner and T. Hokfelt (1998). "Galanin-5-hydroxytryptamine interactions: electrophysiological, immunohistochemical and in situ hybridization studies on rat dorsal raphe neurons with a note on galanin R1 and R2 receptors." Neuroscience **87**(1): 79-94.
